# Volume comparison for $\mathcal {C}^{1,1}$-metrics

**DOI:** 10.1007/s10455-016-9508-2

**Published:** 2016-04-07

**Authors:** Melanie Graf

**Affiliations:** Faculty of Mathematics, University of Vienna, Oskar-Morgenstern-Platz 1, 1090 Vienna, Austria

**Keywords:** Lorentzian manifolds, Riemannian manifolds, Comparison geometry, Low regularity, Singularity theorems, 53C20, 53C50, 83C75

## Abstract

The aim of this paper is to generalize certain volume comparison theorems (Bishop-Gromov and a recent result of Treude and Grant, Ann Global Anal Geom, 43:233–251, 2013) for smooth Riemannian or Lorentzian manifolds to metrics that are only $\mathcal {C}^{1,1}$ (differentiable with Lipschitz continuous derivatives). In particular we establish (using approximation methods) a volume monotonicity result for the evolution of a compact subset of a spacelike, acausal, future causally complete (i.e., the intersection of any past causal cone with the hypersurface is relatively compact) hypersurface with an upper bound on the mean curvature in a globally hyperbolic spacetime with a $\mathcal {C}^{1,1}$-metric with a lower bound on the timelike Ricci curvature, provided all timelike geodesics starting in this compact set exist long enough. As an intermediate step, we also show that the cut locus of such a hypersurface still has measure zero in this regularity—generalizing the well-known result for smooth metrics. To show that these volume comparison results have some very nice applications, we then give a proof of Myers’ theorem, of a simple singularity theorem for globally hyperbolic spacetimes, and of Hawking’s singularity theorem directly in this regularity.

## Introduction

There are many similarities between the ideas used in the proof of Riemannian comparison theorems (in particular Myers’ theorem) and the singularity theorems in Lorentzian geometry. Both use curvature conditions to obtain that in some sense the maximal length of a geodesic without conjugate points is bounded: in the case of Myers’ theorem, one assumes completeness and obtains a bound on the diameter of the manifold (as the distance between two points is given by the length of a minimizing geodesic, which can not have conjugate points) and in the case of, e.g., the Hawking singularity theorem, the assumptions together with geodesic completeness would imply compactness of a certain Cauchy horizon which then gives a contradiction. While there has been some interest in developing Lorentzian analogues to many results from Riemannian comparison geometry in general (see e.g. [1, 2, 10]) this close connection to the singularity theorems was explored further by Treude and Grant in their recent paper [25], where they use Riccati comparison techniques to prove area and volume monotonicity theorems in Lorentzian geometry (with respect to fixed Lorentzian warped product manifolds). These are then applied to give a new proof of the classical Hawking singularity theorem.

We will show that many of these results carry over to $\mathcal {C}^{1,1}$ (locally Lipschitz continuous first derivatives) regularity by showing volume monotonicity results for both Riemannian and Lorentzian $\mathcal {C}^{1,1}$-metrics with appropriate curvature bounds and applying them to prove a version of Myers’ theorem and Hawking’s singularity theorem, respectively.

In general, for a (semi-)Riemannian metric, the class $\mathcal {C}^{1,1}$ is the lowest differentiability class of the metric where one still has local existence and uniqueness of solutions of the geodesic equation. Also by Rademacher’s theorem, all curvature terms still exist almost everywhere and are locally bounded, which allows the definition of curvature bounds in the following way. We say that the Ricci curvature tensor $\mathbf {Ric}$ is bounded from below (by $\kappa$) if for every smooth, local vector field $X\in \mathfrak {X}(U)$ for some open and relatively compact $U\subset M$ one has that the function

1 $$\begin{aligned} p\mapsto \mathbf {Ric}(p)(X_{p},X_{p})-(n-1)\kappa g(p)(X_{p},X_{p}) \end{aligned}$$

is non-negative as an element of $L^{\infty }(U)$ (i.e., is non-negative almost everywhere). If *M* is Lorentzian, we say that the timelike Ricci curvature is bounded from below (by $-\kappa$) if the above holds for any smooth, local timelike vector field. Clearly this coincides with the usual notion for smooth metrics.

As further motivation for studying metrics of this regularity, we give a brief overview about the specific situations in the Riemannian and the Lorentzian setting.

In Riemannian geometry there are ways to generalize curvature bounds to even lower regularity, however this requires—at first glance—very different definitions (see, e.g., [16, 24], where metric measure spaces with lower bounds on the Ricci curvature are studied). While these definitions are equivalent for smooth metrics this has not yet been shown for $\mathcal {C}^{1,1}$-metrics, so at least for now those two approaches are independent.

In Lorentzian geometry, there has recently been an increased interest and many advances in the understanding of low regularity spacetimes (i.e. $\mathcal {C}^{1,1}$- instead of $\mathcal {C}^{2}$-metrics, see [7, 12, 13, 18]), which allowed the proof of both the Hawking and the Penrose singularity theorem in this regularity (see [14, 15]), a problem that had been open for a long time (cf. [22]). From the viewpoint of general relativity, the importance of this regularity is that it allows for a finite jump in the matter variables via the Einstein equations. It is also worth noting that many of the standard results fail dramatically when lowering the regularity further, for example it is shown in [7] that for any $\alpha \in (0,1)$ there exist ‘bubbling metrics’ (of regularity $\mathcal {C}^{0,\alpha }$), whose lightcones have nonempty interior.

The plan of the paper is as follows. In Sect. 2 we study Riemannian manifolds with $\mathcal {C}^{1,1}$-metrics with a lower bound on the Ricci curvature and show a $\mathcal {C}^{1,1}$ version of the Bishop-Gromov volume comparison theorem for Riemannian manifolds with a lower bound on the Ricci curvature. This also serves as a preparation for the Lorentzian case as it requires significantly less technical details but the ideas remain largely the same. In section 3 we first give the definition of the cosmological comparison condition (as introduced in [25]) and a brief overview of relevant results from causality theory for $\mathcal {C}^{1,1}$-metrics, in particular concerning global hyperbolicity and maximizing geodesics to a subset. Then we show the existence of suitable approximating metrics (using results from [7, 13, 14]) and in section 3.3 we show that for $\mathcal {C}^{1,1}$-metrics the cut locus still has measure zero. As a last preparation, we define our comparison spacetimes (again introduced in [25]) as Robertson-Walker spacetimes with constant Ricci curvature and study their dependence on the curvature quantities $\kappa$ and $\beta$. This then allows us to show (as a generalization of [25, Thm. 9] to $\mathcal {C}^{1,1}$-metrics)

### **Theorem 1.1**

(Volume comparison) Let $\kappa ,\beta \in \mathbb {R}$, $g\in \mathcal {C}^{1,1}$ and assume $\left( M,g,\Sigma \right)$ is globally hyperbolic and satisfies $CCC(\kappa ,\beta )$ (see Def. 3.9). Let $A\subset \Sigma$ be compact with $\mu _{\Sigma }(\partial A)=0$, $B\subset \Sigma _{\kappa ,\beta }$ (with finite, non-zero area) and $T>0$ such that all timelike, future directed, unit-speed geodesics starting orthogonally to *A* exist until at least *T*. Then the function

$$\begin{aligned} t\mapsto \frac{\mathrm {vol}\, B_{A}^{+}(t)}{\mathrm {vol}_{\kappa ,\beta }B_{B}^{+}(t)} \end{aligned}$$

is nonincreasing on $\left[ 0,T\right]$.

Finally, in Sect. 4, as applications we give a proof of a $\mathcal {C}^{1,1}$-Myers’ theorem in the Riemannian and two $\mathcal {C}^{1,1}$-singularity theorems (one of them being an alternative proof of the $\mathcal {C}^{1,1}$-version of Hawking’s theorem proved in [14, Thm. 1.1]) in the Lorentzian case.

### **Notation**

Throughout *M* will always be a connected, Hausdorff and second countable smooth manifold of dimension $n\ge 2$. For a semi-Riemannian metric *g* on *M* the curvature tensor of the metric is defined with the convention $R(X,Y)Z=\left( \left[ \nabla _{X},\nabla _{Y}\right] -\nabla _{[X,Y]}\right) Z$ and we denote the Ricci tensor of *g* by $\mathbf {Ric}$.

## Volume comparison for Riemannian $\mathcal {C}^{1,1}$-metrics

The goal of this first section is to show a $\mathcal {C}^{1,1}$ version of the Bishop-Gromov volume comparison theorem.

### **Theorem 2.1**

(Bishop-Gromov) Suppose $\left( M,g\right)$ (with *g* smooth) is a complete Riemannian manifold with $\mathbf {Ric}\ge (n-1)\kappa g$ for some $\kappa \in \mathbb {R}$. Then

$$\begin{aligned} r\mapsto \frac{\mathrm {vol}B_{p}(r)}{\mathrm {vol}_{\kappa }B^{\kappa }(r)}, \end{aligned}$$

where $B^{\kappa }(r)$ denotes any ball of radius *r* in the *n*-dimensional simply connected Riemannian manifold with constant sectional curvature equal to $\kappa$, is a nonincreasing function on $(0,\infty )$ and $\mathrm {vol}B_{p}(r)\le \mathrm {vol}_{\kappa }B^{\kappa }(r)$.

A proof of the classical result (for smooth metrics) can be found, e.g., in [26, Cor. 3.3]. The idea of the proof for $\mathcal {C}^{1,1}$-metrics is to apply the classical result to some smooth approximating metrics, so we first have to show that we can find approximations such that $\left( M,g_{\varepsilon }\right)$ is a complete Riemannian manifold and that for any compact $K\subset M$ and $\delta >0$ we have $\mathbf {Ric}_{\varepsilon }|_{K}\ge \left( n-1\right) (\kappa -\delta )g_{\varepsilon }|_{K}$ (where $\mathbf {Ric}_{\varepsilon }$ denotes the Ricci tensor of $g_{\varepsilon }$) for $\varepsilon$ small enough.

### **Lemma 2.2**

Let $g\in \mathcal {C}^{1,1}$ be a (geodesically) complete Riemannian metric on *M*. Then there exist smooth complete Riemannian metrics $g_{\varepsilon }$ on *M* such that $g_{\varepsilon }\rightarrow g$ in $\mathcal {C}^{1}$, the approximations have locally uniformly bounded second derivatives and

2 $$\begin{aligned} d(g,g_{\varepsilon }):=\sup _{p\in M}\sup _{0\ne X,Y\in T_{p}M}\frac{\left| g(X,Y)-g_{\varepsilon }(X,Y)\right| }{\left| X\right| _{g}\left| Y\right| _{g}}\rightarrow 0. \end{aligned}$$

### *Proof*

It is well known that one can construct smooth, symmetric $\left( 0,2\right)$-tensor fields $\tilde{g}_{\varepsilon }\in \mathcal {T}_{2}^{0}(M)$ with $\tilde{g}_{\varepsilon }\rightarrow g$ in $\mathcal {C}^{1}$ and locally uniformly bounded second derivatives by gluing together componentwise convolutions via a partition of unity: Let $(U_{\alpha },\psi _{\alpha })$ be a (countable) atlas and $\left\{ \chi _{\alpha }\right\}$ a partition of unity subordinate to the $U_{\alpha }$ and choose functions $\zeta _{\alpha }\in \mathcal {C}^{\infty }(U_{\alpha })$ with compact support in $U_{\alpha }$ such that $0\le \zeta _{\alpha }\le 1$ and $\zeta _{\alpha }\equiv 1$ on an open neighborhood of $\mathrm {supp}(\chi _{\alpha })$ in $U_{\alpha }$. Given a locally integrable (*p*, *q*)-tensor field *T* we set

3 $$\begin{aligned} \tilde{T}_{\varepsilon }=\sum _{\alpha }\zeta _{\alpha }\cdot \psi _{\alpha }^{*}\left( \left( \tilde{\chi }_{\alpha }\, T^{\alpha }\right) *\rho _{\varepsilon }\right) , \end{aligned}$$

where $T^{\alpha }\in L_{\mathrm {loc}}^{1}\left( \psi _{\alpha }(U_{\alpha }),\mathbb {R}^{n^{p+q}}\right)$ denotes the chart representation of *T*, $\tilde{\chi }_{\alpha }:=\chi _{\alpha }\circ \psi _{\alpha }^{-1}$ and the convolution is to be understood componentwise. Note that this construction also ensures that the map $\left( \varepsilon ,p\right) \mapsto \tilde{g}_{\varepsilon }(p)$ is smooth.

Now let $\delta >0$. By locally uniform convergence we get that for any $K\subset M$ compact, w.l.o.g. $K\subset U_{\alpha }$ for some chart domain $U_{\alpha }$ (otherwise we may cover *K* by finitely many of those), there exists $\varepsilon _{K}$ such that

4 $$\begin{aligned} \sup _{p\in K}\sup _{0\ne X,Y\in T_{p}M}\frac{\left| g(X,Y)-\tilde{g}_{\varepsilon }(X,Y)\right| }{\left| X\right| _{g}\left| Y\right| _{g}}&\le \sup _{p\in K}\sup _{0\ne X,Y\in T_{p}M}\frac{\left\| \left( g_{ij}-\tilde{g}_{\varepsilon ,ij}\right) X^{j}\right\| _{e}\,\left\| Y\right\| _{e}}{\left| X\right| _{g}\left| Y\right| _{g}} \nonumber \\&\le nC^{2}\sup _{i,j\le n}\sup _{p\in K}\left| g_{ij}(p)-\tilde{g}_{\varepsilon ,ij}(p)\right| <\delta \end{aligned}$$

for all $\varepsilon \le \varepsilon _{K}$ (here $\left\| .\right\| _{e}$ denotes the Euclidean norm on $\mathbb {R}^{n}$ and we used Cauchy’s inequality, $\left\| AX\right\| _{e}\le n\,\max _{i,j\le n}|A_{ij}|\left\| X\right\| _{e}$ and that $\frac{\left\| X\right\| _{e}}{|X|_{g}}<C$, where $C{=}\sup _{\{X\in TM|_{K}:|X|_{g}{=}1\}}\left\| X\right\| _{e}<\infty$, for any $X\in TM|_{K}$). But then the globalization lemma [13, Lem. 2.4] allows us to construct (new) approximations $g_{\varepsilon }:p\mapsto \tilde{g}_{u(\varepsilon ,p)}(p)$ such that for each compact set $K\subset M$ there exists $\varepsilon _{K}$ such that $g_{\varepsilon }(p)=\tilde{g}_{\varepsilon }(p)$ for all $\varepsilon \le \varepsilon _{K}$ and $p\in K$ (in particular the $g_{\varepsilon }$ still satisfy $g_{\varepsilon }\rightarrow g$ in $\mathcal {C}^{1}$ and have locally uniformly bounded second derivatives) and such that for each $\delta >0$ there exists $\varepsilon _{0}(\delta )$ such that $d(g,g_{\varepsilon })<\delta$ for all $\varepsilon \le \varepsilon _{0}$, i.e., $d(g,g_{\varepsilon })\rightarrow 0$.

It remains to show completeness and that the $g_{\varepsilon }$ are Riemannian. This follows from (2.1): For any $\delta >0$ there exists $\varepsilon _{0}$ such that for all $\varepsilon \le \varepsilon _{0}$ one has $\left| g(X,X)-g_{\varepsilon }(X,X)\right| <\delta g(X,X)$ for all $X\in TM$, $X\ne 0$, hence

5 $$\begin{aligned} \left( 1-\delta \right) g(X,X)\le g_{\varepsilon }(X,X)\le \left( 1+\delta \right) g(X,X). \end{aligned}$$

From this, it immediately follows that for $\varepsilon$ small enough positive definiteness of *g* implies positive definiteness of $g_{\varepsilon }$, hence the approximations are Riemannian, and it also immediately gives $\sqrt{1-\delta }L_{g}(\gamma )\le L_{g_{\varepsilon }}(\gamma )\le \sqrt{1+\delta }L_{g}(\gamma )$ for any (locally Lipschitz) curve $\gamma$. But this implies that for $\varepsilon \le \varepsilon _{0}$ we have

6 $$\begin{aligned} \sqrt{1-\delta }\, d_{g}(p,q)\le d_{g_{\varepsilon }}(p,q)\le \sqrt{1+\delta }\ d_{g}(p,q) \end{aligned}$$

and thus $B_{\varepsilon ,p}(r)\subset B_{p}(\frac{r}{\sqrt{1-\delta }})\subset \exp _{p}(\frac{r}{\sqrt{1-\delta }} \cdot \{v\in T_{p}M:\,\left| v\right| _{g}=1\})$ is relatively compact for all $p\in M$ and $r>0$, so $\left( M,g_{\varepsilon }\right)$ is a complete Riemannian manifold by the Hopf-Rinov theorem. $\square$

The next lemma deals with the Ricci curvature estimate and its proof is largely analogous to the Lorentzian version shown in [14, Lem. 3.2] for $\kappa =0$, but a bit less involved.

### **Lemma 2.3**

Let $g\in \mathcal {C}^{1,1}$ be a complete Riemannian metric on *M* that satisfies $\mathbf {Ric}\ge \left( n-1\right) \kappa g$. Then there exist smooth approximations $g_{\varepsilon }$ with all properties of the previous lemma and such that for any compact $K\subset M$ and $\delta >0$ there exists $\varepsilon _{0}$ such that

$$\begin{aligned} \mathbf {Ric}_{\varepsilon }|_{K}\ge \left( n-1\right) \left( \kappa -\delta \right) g_{\varepsilon }|_{K} \end{aligned}$$

for any $$\varepsilon \le \varepsilon _{0}$$.

### *Proof*

We first note that

7 $$\begin{aligned} \mathbf {Ric}_{\varepsilon }-{\tilde{\mathbf{Ric}}}_{\varepsilon }\rightarrow 0\quad \text {uniformly on compact sets}, \end{aligned}$$

where ${\tilde{\mathbf{Ric}}}_{\varepsilon }$ is defined as in (3). This is established by the same arguments as in the proof of [14, Lem. 3.2]: Clearly the only problematic terms are the ones involving second derivatives of the metric (all other terms converge to the respective ones of $\mathbf {Ric}$ in $\mathcal {C}^{0}$). Now on every compact set $g_{\varepsilon }=\tilde{g}_{\varepsilon }$ for $\varepsilon$ small enough by construction, so the terms involving second derivatives of *g* are dealt with using a variant of the Friedrichs lemma, showing that for any $f\in \mathcal {C}^{0}(\mathbb {R}^{n})$ and $g\in L_{\mathrm {loc}}^{\infty }$ the difference $f_{\varepsilon }(h*\rho _{\varepsilon })-(f\, h)*\rho _{\varepsilon }\rightarrow 0$ if $f_{\varepsilon }\rightarrow f$ in $\mathcal {C}^{0}$ (cf. [14, Lem. 3.2]).

Now let $\delta >0$ and $K\subset M$ compact (and w.l.o.g. contained in some chart domain). If we define $A_{\varepsilon }:=\mathbf {Ric}_{\varepsilon }-\left( n-1\right) \kappa g_{\varepsilon }$ and $A:=\mathbf {Ric}-\left( n-1\right) \kappa g$, then clearly also $A_{\varepsilon }-\tilde{A}_{\varepsilon }\rightarrow 0$ uniformly on *K*. So for any $X\in TM|_{K}$

$$\begin{aligned} \left| A_{\varepsilon }(X,X)-\tilde{A}_{\varepsilon }(X,X)\right| \le n\, C^{2}|X|_{g}^{2}\,\sup _{i,j\le n}\sup _{p\in K}\left| A_{\varepsilon ,ij}(p)-\tilde{A}_{\varepsilon ,ij}(p)\right| \le \delta \,(n-1)\, g(X,X) \end{aligned}$$

for $\varepsilon$ small [this follows by similar estimates as in (4)]. So if we can show that $\tilde{A}_{\varepsilon }(X,X)\ge 0$ for all $X\in TM|_{K}$ the claim follows. By construction $\tilde{A}_{\varepsilon }|_{K}$ is a finite sum of terms of the form $\zeta _{\alpha }\psi _{\alpha }^{*}((\tilde{\chi }_{\alpha }A_{ij})*\rho _{\varepsilon })$ (see (3)) so it suffices to show that $\left( (\tilde{\chi }_{\alpha }A_{ij})*\rho _{\varepsilon }\right) (p)$ is a positive semi-definite matrix for any $p\in \psi _{\alpha }(\mathrm {supp}\zeta _{\alpha })$ (note that $(\tilde{\chi }_{\alpha }A_{ij})*\rho _{\varepsilon }$ is well defined on an open neighborhood *U* of $\psi _{\alpha }(\mathrm {supp}\zeta _{\alpha })$ contained in $\psi _{\alpha }(U_{\alpha })$ for $\varepsilon$ small enough). Now let $p\in \psi _{\alpha }(\mathrm {supp}\zeta _{\alpha })$ and $X_{p}\in \mathbb {R}^{n}$ and let $\tilde{X}$ be the constant vector field $x\mapsto X_{p}$ on $\psi _{\alpha }(U_{\alpha })$. Then

$$\begin{aligned} \left( (\tilde{\chi }_{\alpha }A_{ij})*\rho _{\varepsilon }\right) (p)X_{p}^{i}X_{p}^{j}=((\tilde{\chi }_{\alpha }A_{ij}X_{p}^{i}X_{p}^{j})*\rho _{\varepsilon })(p)\ge 0 \end{aligned}$$

since $x\mapsto \tilde{\chi }_{\alpha }(x)A_{ij}(x)\tilde{X}^{i}(x)\tilde{X}^{j}(x)=\tilde{\chi }_{\alpha }(x)A_{ij}(x)X_{p}^{i}X_{p}^{j}$ is non-negative in $L_{\mathrm {loc}}^{\infty }$ by assumption and $\rho _{\varepsilon }\ge 0$. $\square$

These preparations now enable us to show:

### **Theorem 2.4**

(Volume comparison) Let $\left( M,g\right)$ be a complete Riemannian manifold with $g\in \mathcal {C}^{1,1}$ and $\mathbf {Ric}\ge \left( n-1\right) \kappa \, g$. Then

$$\begin{aligned} r\mapsto \frac{\mathrm {vol}B_{p}(r)}{\mathrm {vol}_{\kappa }B^{\kappa }(r)} \end{aligned}$$

is a nonincreasing function on $(0,\infty )$ and $\mathrm {vol}B_{p}(r)\le \mathrm {vol}_{\kappa }B^{\kappa }(r)$.

### *Proof*

Let $p\in M$ and $0<r_{1}<r_{2}<R$. Using the approximating metrics $g_{\varepsilon }$ constructed in Lemma 2.2 and 2.3 we see that for any $\delta >0$ there exists some $\varepsilon _{0}$ such that $\left( B_{p}(R),g_{\varepsilon }\right)$ (as a submanifold of *M*) satisfies the conditions of the classical Bishop-Gromov volume comparison (Thm. 2.1) with $\mathbf {Ric}_{\varepsilon }\ge \left( n-1\right) \left( \kappa -\delta \right) g_{\varepsilon }$ for all $\varepsilon \le \varepsilon _{0}$. This gives us

$$\begin{aligned} 1\ge \frac{\mathrm {vol}_{\varepsilon }B_{p}(r_{1})}{\mathrm {vol}_{\kappa -\delta }B^{\kappa -\delta }(r_{1})}\ge \frac{\mathrm {vol}_{\varepsilon }B_{p}(r_{2})}{\mathrm {vol}_{\kappa -\delta }B^{\kappa -\delta }(r_{2})}. \end{aligned}$$

Now by (6) from the proof of Lemma 2.2 it follows that $d_{g_{\varepsilon }}(p,q)\rightarrow d_{g}(p,q)$ and hence for any $r>0$ one has that $\chi _{B_{\varepsilon ,p}(r)}\rightarrow \chi _{B_{p}(r)}$ almost everywhere (because the sphere $S_{p}(r)\subset \exp _{p}(r\cdot \{v\in T_{p}M:\,\left| v\right| _{g}=1\})$, which has measure zero since $\exp _{p}$ is still locally Lipschitz and $r\cdot \{v\in T_{p}M:\,\left| v\right| _{g}=1\}\subset T_{p}M$ has measure zero). So by dominated convergence (note that $B_{\varepsilon ,p}(r)\subset B_{p}(\frac{r}{\sqrt{1-\delta }})$ by (6) for $\varepsilon$ small, hence the support of all characteristic functions is contained in a common compact set) $\mathrm {vol}_{\varepsilon }B_{p}(r)\rightarrow \mathrm {vol}B_{p}(r)$ for all $r>0$. Calculating the volumes of balls in the comparison spaces shows that $\mathrm {vol}_{\kappa -\delta }B^{\kappa -\delta }(r)=c\,\int _{0}^{r}\mathrm {sn}_{\kappa -\delta }(s)^{n-1}ds\rightarrow c\,\int _{0}^{r}\mathrm {sn}_{\kappa }(s)^{n-1}ds=\mathrm {vol}_{\kappa }B^{\kappa }(r)$, where

$$\begin{aligned} \mathrm {sn}_{\kappa }(s):= {\left\{ \begin{array}{ll} \frac{1}{\sqrt{\kappa }}\sin (\sqrt{\kappa }s) &{} \kappa >0\\ s &{} \kappa =0\\ \frac{1}{\sqrt{|\kappa |}}\sinh (\sqrt{|\kappa |}s) &{} \kappa <0, \end{array}\right. } \end{aligned}$$

for $\delta \rightarrow 0$. Altogether this proves the theorem. $\square$

## The Lorentzian case

In this section the goal is to use volume comparison results (as developed in [25]) for smooth, globally hyperbolic spacetimes *M* with timelike Ricci curvature bounded from below and containing a spacelike hypersurface $\Sigma$ (satisfying some additional causality and completeness conditions) that has mean curvature bounded from above to establish analogous results for $\mathcal {C}^{1,1}$-metrics. It should be noted that these conditions are very similar to those of the Hawking singularity theorem and [25] includes proofs of this theorem using the new comparison techniques therein. So one of the motivations of this paper was to also give an alternative proof of Hawking’s singularity theorem in $\mathcal {C}^{1,1}$-regularity (which was first shown in [14]). This will be done in Sect. 4.2.

However, there are some additional difficulties (compared to the Riemannian result from the previous section) arising due to the metric being Lorentzian: First, one has to be more careful when choosing approximating metrics and simple convolution is no longer sufficient since it need not preserve the causal structure. Here the pioneering work was done by Chruściel and Grant in [7], and from there on causality theory for $\mathcal {C}^{1,1}$ metrics has been developed (see, e.g., [13, 14, 18]). Additionally, the concept of global hyperbolicity for continuous metrics has recently been explored in [21]. This will be helpful in establishing certain results from causality theory for globally hyperbolic spacetimes with a $\mathcal {C}^{1,1}$-metric in Sect. 3.1.

Second, while there is no assumption of (geodesic) completeness needed for the smooth result, an assumption on the minimal time of existence of geodesics starting orthogonally to the hypersurface with unit speed has to be made to ensure that everything plays out in relatively compact sets.

Third, showing that the volumes of the balls in the approximating metrics actually converge to the volumes in the $\mathcal {C}^{1,1}$-metric is a bit more involved and will need a result regarding the cut locus of $\Sigma$ with respect to the $\mathcal {C}^{1,1}$-metric, namely that it has measure zero. This will be shown in Sect. 3.3.

### Basic definitions and results

Throughout this section *M* will always be a Lorentzian manifold with a time orientation. While we will generally assume $\mathcal {C}^{1,1}$ regularity of the metric, we will often include this assumption explicitly to highlight its importance (many of our results will be both well-known in higher and not true, or at least unproven, in lower regularity). We also fix once and for all a (complete) Riemannian background metric *h* on *M*.

As in, e.g., [3, 6] we define causal (timelike) curves to be locally Lipschitz continuous maps $\gamma :I\rightarrow M$ (*I* being an interval) with $\dot{\gamma }\ne 0$ and $g(\dot{\gamma },\dot{\gamma })\le 0$ ($<0$) almost everywhere. A causal curve is called future (past) directed if $\dot{\gamma }$ is future (past) pointing almost everywhere.

For $p,q\in M$ we write $p\ll q$ if there exists a future directed (f.d.) timelike curve from *p* to *q* and $p\le q$ if either $p=q$ or there exists a f.d. causal curve from *p* to *q*. We also define

$$\begin{aligned} I^{+}(p):&=\left\{ q\in M:\, p\ll q\right\} \\ J^{+}(p):&=\{q\in M:\, p\le q\}. \end{aligned}$$

$I^{-}$ and $J^{-}$ are defined analogously. Note that for a $\mathcal {C}^{1,1}$-metric it does not matter whether one allows Lipschitz causal curves or one requires causal curves to be piecewise $\mathcal {C}^{1}$ (or even broken geodesics) in the definition of $I^{+}$ and $J^{+}$ (see [18, Thm.1.27] or [13, Cor.3.10]). Note also that most results from smooth causality theory carry over to $\mathcal {C}^{1,1}$-metrics, we refer to [13, 18] and [14, Appendix A] for an overview.

We will mainly work with globally hyperbolic manifolds and as for smooth metrics one may use any of the following equivalent properties as definition.

#### **Proposition 3.1**

(Global hyperbolicity) Let $\left( M,g\right)$ be a spacetime with $\mathcal {C}^{1,1}$-metric *g*. Then the following properties are equivalent:

1. $\left( M,g\right)$ is causal and for all $p,q\in M$ the set $J(p,q):=J^{+}(p)\cap J^{-}(q)$ is compact,
2. there exists a Cauchy hypersurface *S* for *M* (i.e. a set $S\subset M$ that is met exactly once by every inextendible timelike curve) and
3. $\left( M,g\right)$ is causal and *C*(*p*, *q*) (the space of equivalence classes of future directed causal curves from *p* to *q* with the compact-open topology) is compact

If any of these conditions holds, we say that $\left( M,g\right)$ is globally hyperbolic.

#### *Proof*

In [21], it was shown that these are equivalent even for continuous metrics, if one replaces causality with the slightly stronger assumption of $\left( M,g\right)$ being non-totally imprisoning. So it only remains to show that for a $\mathcal {C}^{1,1}$-metric both (1) and (3) already imply *M* being non-totally imprisoning. This follows as for smooth metrics so we will only present a brief outline: From compactness of *J*(*p*, *q*) (respectively *C*(*p*, *q*)) one obtains that $J^{\pm }(p)$ is closed for all *p*, see [19, Prop. 3.71], respectively [21, Prop. 3.3] (note that the proof only actually uses compactness of *C*(*p*, *q*)). Since $g\in \mathcal {C}^{1,1}$ one can still use the exponential map to show that then already $J^{\pm }(p)=\overline{I^{\pm }(p)}$ [13, Cor. 3.16]. Thus $\left( M,g\right)$ is distinguishing and reflective [19, Prop. 3.64 and 3.65], hence strongly causal (Prop. 3.41, 3.47 and Thm. 3.51 in [19] show the existence of a time function and Prop. 3.57 gives strong causality). That strong causality is stronger than non-totally imprisoning follows again as in the smooth case (see e.g. [20, Lem. 14.13]) as was already remarked in [15]. $\square$

#### *Remark 3.2*

The previous proof also shows that for $\mathcal {C}^{1,1}$-metrics this definition of global hyperbolicity is equivalent to the one in [21].

#### **Definition 3.3**

(Future time separation) Let $p\in M$. Then for $q\in M$ the (future) time separation to *p* is defined by

8 $$\begin{aligned} \tau (p,q):=\sup (\left\{ L(\gamma ):\gamma \,\text {is a f.d. causal curve form }p\text { to }q\right\} \cup \{0\}), \end{aligned}$$

where $L(\gamma )$ denotes the Lorentzian arc-length of $\gamma$, i.e., for a curve $\gamma :(t_1,t_2)\rightarrow M$ one has $L(\gamma ):=\int _{t_1}^{t_2}\sqrt{|g(\dot{\gamma (t)},\dot{\gamma (t)})|}dt$. Similarly one defines the future time separation to a subset $\Sigma$ by

9 $$\begin{aligned} \tau _{\Sigma }(p):=\sup _{q\in \Sigma }\tau (q,p). \end{aligned}$$

If *M* is globally hyperbolic with a continuous metric then any two causally related points can be connected by a maximizing curve [21, Prop. 6.4], hence the supremum in definition (3.1) is attained, so $\tau :M\times M\rightarrow \left[ 0,\infty \right]$ is finite-valued. It is also lower semi-continuous (this holds even if *M* is not globally hyperbolic, see [14, Lem. A.16]). We want to show a similar statement for the time separation to a subset $\Sigma$. This requires some additional properties of $\Sigma$ ([25, Def. 2]).

#### **Definition 3.4**

(Future causally complete) A subset $\Sigma \subset M$ is called *future causally complete* (FCC) if for any $p\in J^{+}(\Sigma )$ the set $J^{-}(p)\cap \Sigma$ has compact relative closure in $\Sigma$.

#### *Remark 3.5*

In a globally hyperbolic manifold the sets $J^{\pm }(p)$ are closed [21, Prop. 3.3] and hence for any FCC subset $\Sigma$ and $p\in J^{+}(\Sigma )$ we have that $J^{-}(p)\cap \Sigma$ is compact and $\Sigma$ itself is closed. Furthermore, from [21, Cor. 3.4], it then follows that

10 $$\begin{aligned} J^{-}(p)\cap J^{+}(J^{-}(p)\cap \Sigma )\quad \mathrm {is\, compact.} \end{aligned}$$

As a preparation for Prop. 3.7 we prove the following limit-curve lemma (that will also be needed again later on), which is a slight modification of Thm. 1.5 in [21] (which is in turn based on [17]):

#### **Lemma 3.6**

Let *M* be globally hyperbolic and $\gamma _{n}:\left[ 0,1\right] \rightarrow M$ be a sequence of causal curves and $K\subset M$ compact such that $\gamma _{n}\subset K$ for all $n\in \mathbb {N}$. Then there exists a subsequence $\gamma _{n_{k}}$ that converges (*h*-)uniformly to a causal curve $\gamma :\left[ 0,1\right] \rightarrow M$ (i.e. $\sup _{t\in \left[ 0,1\right] }d_{h}(\gamma _{n_{k}}(t),\gamma (t))\rightarrow 0$) with

11 $$\begin{aligned} L(\gamma )\ge \limsup _{k\rightarrow \infty }L(\gamma _{n_{k}}). \end{aligned}$$

In particular, if the $\gamma _{n}$ are maximizing, then $\gamma$ is as well.

#### *Proof*

By [21, Lem. 2.7] we get an upper bound on the Lipschitz constants of the $\gamma _{n}$. And so, since the sequence must have an accumulation point, the convergence result follows from Thm. 1.5 of [21].

It remains to show (11) and that $\gamma$ is maximizing if the $\gamma _{n}$ are. By [21, Thm. 6.3] the length functional $L:\left\{ \gamma \in \mathcal {C}\left( \left[ 0,1\right] ,K\right) :\,\gamma \,\text {causal}\right\} \rightarrow [0,\infty )$ is upper semi-continuous w.r.t. *h*-uniform convergence as defined above (note that while the statement there only deals with a special subset of causal curves defined on $\left[ 0,1\right]$, the proof works for any set of such curves with an upper bound on the Lipschitz constants), so $L(\gamma )\ge \limsup L(\gamma _{n_{k}})$. Using this and lower semi-continuity of $\tau$ (see [14, Lem. A.16]) gives

$$\begin{aligned} L(\gamma )\ge \limsup L(\gamma _{n_{k}})=\limsup \tau (\gamma _{n_{k}}(0),\gamma _{n_{k}}(1))\ge \tau \left( \gamma (0),\gamma (1)\right) , \end{aligned}$$

so $\gamma$ is maximizing. $\square$

For an acausal, spacelike FCC hypersurface in a globally hyperbolic manifold the following holds (which is shown largely analogous to the smooth case ([25, Thm. 2]), only using Lemma 3.6 instead of other limit curve results, we nevertheless include a complete proof):

#### **Proposition 3.7**

Let $\left( M,g\right)$ with $g\in \mathcal {C}^{1,1}$ be globally hyperbolic and let $\Sigma \subset M$ be an acausal, FCC subset. Then the future time separation $\tau _{\Sigma }$ to $\Sigma$ is finite-valued and continuous on *M* and for any $p\in J^{+}(\Sigma )\setminus \Sigma$ there exists $q\in \Sigma$ and a causal curve $\gamma$ from *q* to *p* with $\tau _{\Sigma }(p)=\tau (q,p)=L(\gamma )$. Any such maximizing curve $\gamma$ has to be a (reparametrization of) a geodesic, which is timelike for $p\in I^{+}(\Sigma )$ and null otherwise. If $\Sigma \subset M$ is, additionally, a spacelike hypersurface, then for $p\in I^{+}(\Sigma )$ any maximizing geodesic has to start orthogonally to $\Sigma$.

#### *Proof*

If $p\notin I^{+}(\Sigma )$ then $\tau _{\Sigma }(p)=0$. Now let $p\in J^{+}(\Sigma )\setminus \Sigma$. Then there exists a causal curve $\gamma$ from *p* to $q\in \Sigma$ and if $p\notin I^{+}(\Sigma )$ then clearly $L(\gamma )\le \tau _{\Sigma }(p)=0\le L(\gamma )$. So assume $p\in I^{+}(\Sigma )$. By definition of $\tau _{\Sigma }$ there exist $q_{n}\in \Sigma$ such that $\tau (q_{n},p)\rightarrow \tau _{\Sigma }(p)$. Since $p\in I^{+}(\Sigma )$ we have $\tau _{\Sigma }(p)>0$ and hence $\tau (q_{n},p)>0$ for *n* large, so $q_{n}$ and *p* are causally related and can be connected by a maximizing curve $\gamma _{n}$ (see [21, Prop. 6.4]). Because $q_{n}\in J^{-}(p)\cap \Sigma$, all the $\gamma _{n}$ are contained in $J^{-}(p)\cap J^{+}(J^{-}(p)\cap \Sigma )$, which is compact by Remark 3.5. Therefore (after maybe reparametrizing and passing to a subsequence), Lemma 3.6 gives a uniform limit curve $\gamma$ that is causal, satisfies $q=\gamma (0)\in \Sigma$ (note that $\Sigma$ is closed by Remark 3.5) and $p=\gamma (1)$ and is maximizing, so by upper semi-continuity of the length functional we get

$$\begin{aligned} \tau (p,q)=L(\gamma )\ge \limsup L(\gamma _{n})=\limsup \tau (q_{n},p)=\tau _{\Sigma }(p). \end{aligned}$$

Consequently, $\gamma$ maximizes the distance from $\Sigma$ to *p* and $\tau _{\Sigma }(p)$ is finite.

Regarding continuity we show lower and upper semi-continuity separately, starting with lower semi-continuity. Let $p\in M$. We have to show that for every $\varepsilon$ there exists a neighborhood $U_{\varepsilon }$ of *p* such that for all $q\in U_{\varepsilon }$

$$\begin{aligned} \tau _{\Sigma }(q)\ge \tau _{\Sigma }(p)-\varepsilon . \end{aligned}$$

If $\tau _{\Sigma }(p)=0$, there is nothing to prove due to non-negativity of $\tau _{\Sigma }$. Let $\gamma :\left[ 0,1\right] \rightarrow M$ be a causal curve from $p_{0}\in \Sigma$ to *p* such that $L(\gamma )=\tau (p_{\text {0}},p)=\tau _{\Sigma }(p)>0$. Now for any $\varepsilon >0$ there exists $t_{\varepsilon }$ such that $L(\gamma |_{\left[ t_{\varepsilon },1\right] })<\varepsilon$. Then $U_{\varepsilon }:=I^{+}(\gamma (t_{\varepsilon }))$ is a neighborhood of *p* such that for all $q\in U_{\varepsilon }$

$$\begin{aligned} \tau _{\Sigma }(q)\ge L(\gamma |_{\left[ 0,t_{\varepsilon }\right] })=\tau _{\Sigma }(p)-L(\gamma |_{\left[ t_{\varepsilon },1\right] })\ge \tau _{\Sigma }(p)-\varepsilon . \end{aligned}$$

Next we show upper semi-continuity, i.e., for every $\varepsilon$ there exists a neighborhood $U_{\varepsilon }$ of *p* such that for all $q\in U_{\varepsilon }$

$$\begin{aligned} \tau _{\Sigma }(q)\le \tau _{\Sigma }(p)+\varepsilon . \end{aligned}$$

Assume to the contrary that there exists $\varepsilon >0$ and $p_{n}\rightarrow p$ such that

$$\begin{aligned} \tau _{\Sigma }(p_{n})>\tau _{\Sigma }(p)+\varepsilon \end{aligned}$$

and let $\gamma _{p_{n}}:\left[ 0,1\right] \rightarrow M$ be causal curves from $\Sigma$ to $p_{n}$ with $\tau _{\Sigma }(p_{n})=L(\gamma _{p_{n}})$ (such curves exist, since $\tau _{\Sigma }(p_{n})>\tau _{\Sigma }(p)+\varepsilon >0$ and so $p_{n}\in I^{+}(\Sigma )$). Let $p^{+}\in I^{+}(p)$, then $p_{n}\in J^{-}(p^{+})$ eventually and thus $\gamma _{p_{n}}\subset J^{-}(p^{+})\cap J^{+}(J^{-}(p^{+})\cap \Sigma )$, which is compact by Remark 3.5. So, we can apply Lemma 3.6 to obtain (after passing to a subsequence) a curve $\gamma$ from $\Sigma$ to $p=\lim p_{n}$ with

$$\begin{aligned} \tau _{\Sigma }(p)\ge L(\gamma )\ge \limsup _{n\rightarrow \infty }L(\gamma _{p_{n}})=\limsup _{n\rightarrow \infty }\tau _{\Sigma }(p_{n})\ge \tau _{\Sigma }(p)+\varepsilon \end{aligned}$$

which is a contradiction.

Since causal geodesics are locally maximizing (by [18, Thm. 6]), any maximizing curve must be (a reparametrization of) a geodesic and if $p\in I^{+}(\Sigma )$ then $\tau _{\Sigma }(p)>0$, so it has to be timelike.

Now let $\Sigma$ be an acausal, FCC, spacelike hypersurface. We show that all timelike geodesics that start in $\Sigma$ and maximize the distance to $\Sigma$ must start orthogonally: First note that if $\gamma :\left[ 0,b\right] \rightarrow M$ maximizes the distance then also $\gamma |_{\left[ 0,\varepsilon \right] }$ must maximize the distance to $\Sigma$, so this is a local question and we may assume that $M=\mathbb {R}^{n}$, $\Sigma \subset \mathbb {R}^{n}$ is a hypersurface and $\gamma :\left[ 0,1\right] \rightarrow \mathbb {R}^{n}$ is a timelike unit-speed geodesic with $\gamma (0)=0\in \Sigma$ that maximizes the distance to $\Sigma$. Now for any $v\in T_{0}\Sigma$ we can find a smooth curve $\alpha :\left[ 0,\varepsilon \right] \rightarrow \Sigma$ such that $\dot{\alpha }(0)=v$ and $\alpha (0)=0$. We use this to define a $\mathcal {C}^{2,1}$ (note that $\gamma$ is a geodesic, hence $\mathcal {C}^{2,1}$ by the geodesic equation) variation

$$\begin{aligned} \sigma :\left[ 0,1\right] \times \left[ 0,\varepsilon \right]&\rightarrow \mathbb {R}^{n}\\ \sigma (t,s)&=\gamma (t)+(1-t)\alpha (s). \end{aligned}$$

Since $\gamma$ is timelike, this is a timelike variation for small enough $\varepsilon$ and we may use the first variation of arc-length (see [20, Prop. 10.2] and note that $s\mapsto L(\sigma (.,s))$ is still $\mathcal {C}^{1}$) to obtain

$$\begin{aligned} 0=L'(0)=\int _{0}^{1}g(\ddot{\gamma }(t),(\partial _{s}\sigma )(t,0))dt+g(v,\dot{\gamma }(0))=g(v,\dot{\gamma }(0)). \end{aligned}$$

This shows that $\dot{\gamma }(0)\perp v$ for all $v\in T_{0}\Sigma$, so $\gamma$ starts orthogonally. $\square$

Note that the part of the proof that shows that $\gamma$ has to start orthogonally to $\Sigma$ really only works for $p\in I^{+}(\Sigma )$ and not for $p\in J^{+}(\Sigma )$ since in that case one could not guarantee that the constructed variation consists only of timelike curves. However, the next remark shows that $J^{+}(\Sigma )\setminus \left( \Sigma \cup I^{+}(\Sigma )\right) =\emptyset$ anyways.

#### *Remark 3.8*

If $\Sigma$ is an acausal, FCC hypersurface then actually $J^{+}(\Sigma )\setminus \Sigma =I^{+}(\Sigma )$. The argument is the same as for smooth metrics: First, any FCC set must be closed (by Remark 3.5) and then [20, Cor. 14.26] shows that $\mathrm {edge}(\Sigma )=\emptyset$. By Proposition 3.7 any $p\in J^{+}(\Sigma )\setminus \left( \Sigma \cup I^{+}(\Sigma )\right)$ is the future endpoint of a lightlike geodesic $\gamma$ starting in $\gamma (0)\in \Sigma$. Now if $\gamma (0)\notin \mathrm {edge}(\Sigma )$ then for $\varepsilon$ small enough $\gamma (\varepsilon )\in I^{+}(\Sigma )$ (since by definition of $\mathrm {edge}(\Sigma )$ [20, Def. 14.23] there must exist a $q^{-}\in I^{-}(\gamma (0))$ such that any timelike curve connecting $q^{-}$ to $\gamma (\varepsilon )$ meets $\Sigma$) contradicting $p\notin I^{+}(\Sigma )$. But since $\mathrm {edge}(\Sigma )=\emptyset$ this shows that $J^{+}(\Sigma )\setminus \left( \Sigma \cup I^{+}(\Sigma )\right) =\emptyset$.

Finally, we will specify the curvature conditions, introduced in [25, Def. 5], $\left( M,g\right)$ has to satisfy for the volume comparison theorem (Theorem 1.1) we are going to show.

#### **Definition 3.9**

*(Cosmological comparison condition)* Let $\kappa ,\beta \in \mathbb {R}$. We say that a spacetime $\left( M,g,\Sigma \right)$ satisfies the cosmological comparison condition $CCC(\kappa ,\beta )$ if

1. $\Sigma \subset M$ is a smooth, spacelike, acausal, FCC hypersurface and the mean curvature *H* of $\Sigma$ satisfies $H\le \beta$ and
2. $\mathbf {Ric}(X,X)\ge -\left( n-1\right) \kappa \, g(X,X)$ in $L_{\mathrm {loc}}^{\infty }$ for any local, smooth timelike vector field *X* (i.e., the timelike Ricci curvature is bounded from below by $\kappa$ in the sense of (1))

#### *Remark 3.10*

Following [20] our sign conventions regarding the mean curvature are as follows: Let $\Sigma$ be a spacelike hypersurface and $\mathbf {n}$ be the f.d. timelike unit normal vector field to $\Sigma$. We define the shape operator $S_{\mathbf {n}}:T\Sigma \rightarrow T\Sigma$ of $\Sigma$ by $S_{\mathbf {n}}(V):=\mathrm {tan}_{\Sigma }\nabla _{V}\mathbf {n}$, where $\mathrm {tan}_{\Sigma }$ denotes the tangential projection $TM|_{\Sigma }\rightarrow T\Sigma$. Using the shape operator we can write the mean curvature as $H=\mathrm {tr}_{g|_{T\Sigma }}\, S_{\mathbf {n}}$, where $g|_{T\Sigma }$ denotes the metric on $\Sigma$ induced by *g*.

Note that even though basically all of the upcoming results (except for the $\mathcal {C}^{1,1}$ version of Hawking’s theorem at the very end, see Theorem 4.5) will additionally require global hyperbolicity, we choose not to include this in the definition of the comparison condition.

### Construction and properties of the approximating metrics

We need to establish some properties of suitable approximations for a $\mathcal {C}^{1,1}$-metric *g* with a hypersurface $\Sigma$ satisfying $CCC(\kappa ,\beta )$. This is done in the following three lemmas. To start with, we use approximations as constructed in [7], i.e., we have (using the formulation of [13, Prop. 2.5]):

#### **Proposition 3.11**

Let $\left( M,g\right)$ be a spacetime with a continuous Lorentzian metric, and *h* some smooth background Riemannian metric on M. Then for any $\varepsilon >0$, there exist smooth Lorentzian metrics $\check{g}_{\varepsilon }$ and $\hat{g}_{\varepsilon }$ on *M* such that $\check{g}_{\varepsilon }\prec g\prec \hat{g}_{\varepsilon }$, i.e.

$$\begin{aligned} \forall X\in TM:\,\check{g}_{\varepsilon }(X,X)\le 0\implies g(X,X)<0\,\mathrm {and}\, g(X,X)\le 0\implies \hat{g}_{\varepsilon }(X,X)<0, \end{aligned}$$

and $$d_{h}(\check{g}_{\varepsilon },g)+d_{h}(\hat{g}_{\varepsilon },g)<\varepsilon $$, where

$$\begin{aligned} d_{h}(g_{1},g_{2}):=\sup _{p\in M}\sup _{0\ne X,Y\in T_{p}M}\frac{\left| g_{1}(X,Y)-g_{2}(X,Y)\right| }{\left\| X\right\| _{h}\left\| Y\right\| _{h}}. \end{aligned}$$

Moreover, $\check{g}_{\varepsilon }$ and $\hat{g}_{\varepsilon }$ depend smoothly on $\varepsilon$, and if $g\in \mathcal {C}^{1,1}$ then $\check{g}_{\varepsilon }$ and $\hat{g}_{\varepsilon }$ additionally satisfy

1. They converge to *g* in the $\mathcal {C}^{1}$-topology as $\varepsilon \rightarrow 0$ and
2. The second derivatives are bounded, uniformly in $\varepsilon$, on compact sets.

Now we show that we may additionally demand the following:

#### **Lemma 3.12**

Let (*M*, *g*) be globally hyperbolic with $g\in \mathcal {C}^{1,1}$ and let $\Sigma \subset M$ be a smooth acausal, spacelike FCC hypersurface. Then there exist smooth approximations $g_{\varepsilon }$ such that the approximations $\left( M,g_{\varepsilon }\right)$ are globally hyperbolic and $\Sigma \subset M$ is a smooth acausal, spacelike FCC hypersurface (w.r.t. $g_{\varepsilon }$).

#### *Proof*

We first show that we can construct approximations $g_{\varepsilon }$ that retain the properties of the $\check{g}_{\varepsilon }$ from above but additionally satisfy that $\Sigma$ is $g_{\varepsilon }$ spacelike for $\varepsilon$ small, i.e. $g_{\varepsilon }|_{T\Sigma }$ is positive definite. To do this, we show that for every compact set $K\subset \Sigma$ there exists $\varepsilon _{K}$ such that this holds for the $\check{g}_{\varepsilon }$ for all $\varepsilon \le \varepsilon _{K}$ and then apply the globalization lemma [13, Lem. 2.4]. This gives us metrics $g_{\varepsilon }(p):=\check{g}_{\tilde{\varepsilon }(\varepsilon ,p)}(p)$ that satisfy $g_{\varepsilon }|_{K}=\check{g}_{\varepsilon }|_{K}$ for all $\varepsilon \le \varepsilon _{K}$ and $g_{\varepsilon }|_{T\Sigma }$ is positive definite. Since $g|_{T\Sigma }$ is a Riemannian metric on $\Sigma$ we have that $g(X,X)=1$ implies $\left\| X\right\| _{h}\le C$ for all $X\in T\Sigma |_{K}$ and hence $\sup _{\{X\in T\Sigma |_{K}:g(X,X)=1\}}\check{g}_{\varepsilon }(X,X)-g(X,X)\rightarrow 0$ by the previous proposition. So $\check{g}_{\varepsilon }(X,X)>c\, g(X,X)>0$ for any nonzero $X\in T\Sigma |_{K}$ for all $\varepsilon$ small (depending on *K*), showing positive definiteness.

The other properties follow because by the above construction $g_{\varepsilon }\prec g$ (since $g_{\varepsilon }(p)=\check{g}_{\tilde{\varepsilon }(\varepsilon ,p)}(p)$ and $\check{g}_{\varepsilon }\prec g$): By Proposition 3.1, global hyperbolicity is equivalent to the existence of a Cauchy hypersurface and by definition any Cauchy hypersurface for *g* also has to be a Cauchy hypersurface for any $g'\prec g$. This shows that $\left( M,g_{\varepsilon }\right)$ is globally hyperbolic. Similarly $\Sigma$ being *g*-FCC implies $g_{\varepsilon }$-FCC and *g*-acausality of $\Sigma$ implies $g_{\varepsilon }$-acausality. $\square$

From now on $g_{\varepsilon }$ will always denote smooth approximating metrics as constructed above, in particular satisfying Proposition 3.11, Lemma 3.12 and $g_{\varepsilon }\prec g$. The next lemma shows properties of the Ricci curvature $\mathbf {Ric}_{\varepsilon }$ of this approximations (which is basically [14, Lem. 3.2], except also explicitly covering the case $\kappa \ne 0$, and the proof proceeds similarly).

#### **Lemma 3.13**

Let $g\in \mathcal {C}^{1,1}$ and *h* be a background Riemannian metric. Suppose that $\mathbf {Ric}_{g}(X,X)\ge -n\,\kappa \, g(X,X)$ for any local smooth *g*-timelike vector field $X\in \mathfrak {X}(U)$. Then for any compact set $K\subset M$, $C>0$ and $\delta >0$ there exists $\varepsilon _{0}=\varepsilon _{0}(K,C,\delta )$ such that

12 $$\begin{aligned} \mathbf {Ric}_{\varepsilon }(X,X)\ge (n-1)\,(\kappa -\delta )\quad \forall X\in TM|_{K}\,\mathrm {with}\, g_{\varepsilon }(X,X)=-1\,\mathrm {and}\,\left\| X\right\| _{h}<C \end{aligned}$$

for all $$\varepsilon <\varepsilon _{0}$$.

#### *Proof*

Fix $K\subset M$ (w.l.o.g. contained in a chart domain), $C>0$ and $\delta >0$. As in the proof of Lemma 2.3 we proceed similarly to [14, Lem. 3.2]. By the argument given there $g_{\varepsilon }-\tilde{g}_{\varepsilon }\rightarrow 0$ in $\mathcal {C}^{2}$ (note that by construction $g_{\varepsilon }=\check{g}_{\varepsilon }$ on *K* for $\varepsilon$ small). As in (7) we have $\mathbf {Ric}_{\tilde{g}_{\varepsilon }}-\tilde{\mathbf {Ric}}_{\varepsilon }\rightarrow 0$ uniformly on compact sets and so

13 $$\begin{aligned} \mathbf {Ric}_{\varepsilon }-\tilde{\mathbf {Ric}}_{\varepsilon }\rightarrow 0\quad \text {uniformly on compact sets.} \end{aligned}$$

Now we define $A_{\varepsilon }:=\mathbf {Ric}_{\varepsilon }-\left( n-1\right) \kappa g_{\varepsilon }$ and $A:=\mathbf {Ric}-\left( n-1\right) \kappa g$. Clearly $A_{\varepsilon }-\tilde{A}_{\varepsilon }\rightarrow 0$ uniformly on compact sets and thus (for $\varepsilon$ small enough)

$$\begin{aligned} \left| A_{\varepsilon }(X,X)-\tilde{A}_{\varepsilon }(X,X)\right| \le c\,\sup _{i,j\le n}\sup _{p\in K}\left| A_{ij}(p)-\tilde{A}_{\varepsilon ,ij}(p)\right| <\delta (n-1) \end{aligned}$$

for all $X\in TM|_{K}$ with $\left\| X\right\| _{h}\le C$. So if we can show that $\tilde{A}_{\varepsilon }(X,X)\ge 0$ for all $X\in TM|_{K}$ with $\left\| X\right\| _{h}\le C$ and $g_{\varepsilon }(X,X)=-1$ the claim follows.

As in Lemma 2.3 it now suffices to show this for every term of $\tilde{A}_{\varepsilon }$ of the form $\zeta _{\alpha }\psi _{\alpha }^{*}((\tilde{\chi }_{\alpha }A_{ij})*\rho _{\varepsilon })$. Again we may assume $M=\mathbb {R}^{n}$ and $\tilde{A}_{\varepsilon }=A*\rho _{\varepsilon }$. Now choose $\varepsilon _{0}$ such that $\left| g_{\varepsilon }(X,X)-g\left( X,X\right) \right| <\frac{1}{2}$ for all $X\in TM|_{K}$ with $\left\| X\right\| _{h}\le C$ and all $\varepsilon <\varepsilon _{0}$. Since *g* is uniformly continuous on *K* there exists some $\varepsilon _{0}>r>0$ such that for any $p,x\in K$ with $\left\| x-p\right\| _{h}<r$ and any $X_{p}\in T_{p}M=\mathbb {R}^{n}$ with $\left\| X_{p}\right\| _{h}\le C$ we have $\left| g(p)(X_{p},X_{p})-g(x)(X_{p},X_{p})\right| <\frac{1}{2}$. This implies that for any $p\in K$ and $X_{p}\in \mathbb {R}^{n}$ with $\left\| X_{p}\right\| _{h}\le C$ and $g_{\varepsilon }(p)(X_{p},X_{p})=-1$ the constant vector field $\tilde{X}:\, x\mapsto X_{p}$ is *g* timelike on on the open ball $B_{p}(r)$ and thus by our assumption $A(\tilde{X},\tilde{X})=\mathbf {Ric}(\tilde{X},\tilde{X})-\left( n-1\right) \kappa g(\tilde{X},\tilde{X})\ge 0$ almost everywhere on $B_{p}(r)$. So for $\varepsilon <r$ we get

$$\begin{aligned} \tilde{A}_{\varepsilon }(p)(X_{p},X_{p})=(A*\rho _{\varepsilon })(p)(X_{p},X_{p})=(A(\tilde{X},\tilde{X})*\rho _{\varepsilon })(p)\ge 0, \end{aligned}$$

since $$\rho _{\varepsilon }\ge 0$$ and $$\mathrm {supp}\rho _{\varepsilon }\subset B_{0}(\varepsilon )$$. $$\square $$

#### *Remark 3.14*

Note that the condition $\left\| X\right\| _{h}<C$ in the inequality (12) was not necessary in the Riemannian case (see Lemma 2.3) since *g* itself was Riemannian, but is vital for Lorentzian metrics and without it, the result is probably not true: For example, if $M=\mathbb {R}^{3}$ with $g=\mathrm {diag}(-1,1,1)$ and $g_{\varepsilon }=\mathrm {diag}(-1-\varepsilon x^{2}y^{2}z^{2},1,1)$ then $g_{\varepsilon }\rightarrow g$ even in $\mathcal {C}^{\infty }$ and $\mathbf {Ric}_{g}(X,X)\ge 0$, but for $p=(1,1,1)\in M$, $N\in \mathbb {N}$ and any $\varepsilon >0$ there exists $X=X(N,\varepsilon )\in T_{p}M=\mathbb {R}^{3}$ such that $g_{\varepsilon }(p)(X,X)=-1$ but still

$$\begin{aligned} \mathbf {Ric}_{g_{\varepsilon }}(p)(X,X)<-N. \end{aligned}$$

However, these $X(N,\varepsilon )$ do not satisfy $\left\| X(N,\varepsilon )\right\| _{h}<C$ for any $C>0$ independent of $\varepsilon$ and *N*. A straightforward calculation gives

$$\begin{aligned} \mathbf {Ric}_{g_{\varepsilon }}(p)=\frac{1}{\left( 1+\varepsilon \right) ^{2}}\left( \begin{array}{ccc} \left( 1+\varepsilon \right) 2\varepsilon &{} 0 &{} 0\\ 0 &{} -\varepsilon &{} -\varepsilon \left( 2+\varepsilon \right) \\ 0 &{} -\varepsilon \left( 2+\varepsilon \right) &{} -\varepsilon \end{array}\right) . \end{aligned}$$

Now let $X=(x,y,y)\in T_{p}M$ and demand $-1=g_{\varepsilon }(p)(X,X)=\left( -1-\varepsilon \right) x^{2}+2y^{2}$. Then

$$\begin{aligned} \mathbf {Ric}_{g_{\varepsilon }}(p)(X,X)&=\frac{2\varepsilon }{\left( 1+\varepsilon \right) ^{2}}\left[ 1-(1+\varepsilon )y^{2}\right] \end{aligned}$$

which diverges to $-\infty$ as $y\rightarrow \infty$ for any fixed $\varepsilon$.

#### **Lemma 3.15**

Let $g\in \mathcal {C}^{1,1}$ and assume that the mean curvature of $\Sigma \subset M$ is bounded from above by $\beta$. Then there exist approximations $g_{\varepsilon }$ such that for any compact set $A\subset \Sigma$ and $\eta >0$ there exists $\varepsilon _{0}$ such that $H_{\varepsilon }|_{A}<\beta +\eta$ for all $\varepsilon <\varepsilon _{0}$.

#### *Proof*

Since $H=\mathrm {tr}_{g|_{T\Sigma }}\, S_{\mathbf {n}}$ (see Remark 3.10) and the Christoffel symbols of $g_{\varepsilon }$ converge to those of *g* uniformly on compact sets it suffices to show that the the $g_{\varepsilon }$ unit normal vector field $\mathbf {n}_{\varepsilon }$ to $\Sigma$ converges to $\mathbf {n}$ in $\mathcal {C}^{1}$. Because $\Sigma$ is a smooth hypersurface it is locally given as the zero set of a submersion $f:U\rightarrow \mathbb {R}^{n-1}$ and hence

14 $$\begin{aligned} \mathbf {n}_{\varepsilon }=\frac{\mathrm {grad}_{\varepsilon }f}{\left| \mathrm {grad}_{\varepsilon }f\right| _{g_{\varepsilon }}}\rightarrow \frac{\mathrm {grad}f}{\left| \mathrm {grad}f\right| _{g}}=\mathbf {n} \end{aligned}$$

in $\mathcal {C}^{1}$, proving the claim. $\square$

We need two further properties of this approximations.

#### **Proposition 3.16**

Let $K\subset TM$ be compact and $T>0$ such that all *g*-geodesics starting in *K* exist for all $t\le T$. Then there exists $\varepsilon _{0}>0$ such that for all $\varepsilon _{0}\ge \varepsilon \ge 0$ every $g_{\varepsilon }$-geodesic starting in *K* exists until at least time *T* and the function

$$\begin{aligned} f:\left[ 0,\varepsilon _{0}\right] \times \left[ 0,T\right] \times K&\rightarrow TM\\ \left( \varepsilon ,t,v\right)&\mapsto \dot{\gamma }_{v}^{\varepsilon }(t), \end{aligned}$$

where $\gamma _{v}^{\varepsilon }$ denotes the $g_{\varepsilon }$-geodesic with $\dot{\gamma }_{v}^{\varepsilon }(0)=v$, is continuous.

#### *Proof*

This follows from a local argument using a standard result on the comparison of solutions to ODE [8, 10.5.6 and 10.5.6.1]: Note that the $\Gamma _{g,ij}^{k}$ are locally Lipschitz continuous, the $\Gamma _{\varepsilon ,ij}^{k}(p)$ depend smoothly on $\varepsilon$ and *p* for $\varepsilon >0$ and $\Gamma _{\varepsilon ,ij}^{k}\rightarrow \Gamma _{g,ij}^{k}$ locally uniformly for $\varepsilon \rightarrow 0$. Given any $v\in K$ we choose chart domains $U_i \subset TM$ ($i=0,\ldots ,m$) covering $\dot{\gamma }_v([0,T])$ and times $t_i$ such that $\dot{\gamma }_v([t_i,t_{i+1}])\subset U_i$. Let $k^i$ be an upper bound for the Lipschitz constants of the derivatives of *g* and $g_\varepsilon$ in $U_i$ and $\alpha ^i$ be chosen such that $|\Gamma _{\varepsilon ,lj}^{k}-\Gamma _{g,lj}^{k}|<\alpha ^i$ on $U_i$. Then by [8, 10.5.6 and 10.5.6.1] for any $\tilde{v}\in U_0\cap K$ with $\left\| v^0-\tilde{v}^0 \right\| _{e}<\mu ^0$ one has that for $\mu ^0$ and $\alpha ^0$ sufficiently small $\dot{\gamma }^{\varepsilon ,0}$ exists until at least $t_1$ and $\left\| \dot{\gamma }^0_v(t)-\dot{\gamma }^{\varepsilon ,0}_{\tilde{v}}(t)\right\| _{e} \le \mu e^{tk}+\alpha \frac{e^{tk}-1}{k}$ for all $t\in [0,t_1]$. Continuing this in $U_1$ (with initial data $\dot{\gamma }_v(t_1)$ and $\dot{\gamma }^{\varepsilon }_{\tilde{v}}(t_1)$, which will be close if *v* and $\tilde{v}$ were), $U_2$ and so forth gives the claim. $\square$

#### **Definition 3.17**

*(Unit normal bundle)* We write $S^{+}N\Sigma$ (or sometimes also $S_{0}^{+}N_{0}\Sigma$) for the (future) unit normal bundle to $\Sigma$, i.e. 

$$\begin{aligned} S^{+}N\Sigma :=\left\{ v\in TM|_{\Sigma }:\, v\, f.p.,\, g(v,w)=0\,\forall w\in T_{\pi (v)}\Sigma \,\text {and}\, g(v,v)=-1\right\} \subset TM|_{\Sigma } \end{aligned}$$

and analogously $S_{\varepsilon }^{+}N_{\varepsilon }\Sigma$ for the (future) unit normal bundle to $\Sigma$ w.r.t. the metric $g_{\varepsilon }$. For any $A\subset \Sigma$ we further define $S^{+}NA\equiv S_{0}^{+}N_{0}A:=\left\{ v\in S^{+}N\Sigma :\,\pi (v)\in A\right\}$ and analogously $S_{\varepsilon }^{+}N_{\varepsilon }A$.

For compact $A\subset \Sigma$ each $S_{\varepsilon }^{+}N_{\varepsilon }A$ is compact for any $\varepsilon \ge 0$ (since the respective future pointing unit normal vector fields $\mathbf {n}_{\varepsilon }$ are continuous and $S_{\varepsilon }^{+}N_{\varepsilon }A=\mathbf {n}_{\varepsilon }(A)$ by definition). The following lemma shows that this remains true for their union over $0\le \varepsilon \le \varepsilon _{0}$.

#### **Lemma 3.18**

Let $A\subset \Sigma$ be compact. Then for any neighborhood *U* of $S^{+}NA$ in $TM|_{\Sigma }$ there exists $\varepsilon _{0}(U,A)>0$ such that

$$\begin{aligned} \bigcup _{0\le \varepsilon \le \varepsilon _{0}}S_{\varepsilon }^{+}N_{\varepsilon }A\subset U\subset TM|_{\Sigma } \end{aligned}$$

and is compact.

#### *Proof*

By definition of the unit normal bundles $S_{\varepsilon }^{+}N_{\varepsilon }A$ we have $\bigcup _{0\le \varepsilon \le \varepsilon _{0}}S_{\varepsilon }^{+}N_{\varepsilon }A=n([0,\varepsilon _{0}],A)$ where $n:[0,1]\times \Sigma \rightarrow TM|_{\Sigma }$ is defined by $n(\varepsilon ,p):=\mathbf {n}_{\varepsilon }(p)$, so the assertion follows from continuity of *n* (which in turn follows directly from (14)).

### The cut locus of $\Sigma$ has measure zero

As a further preparation we will now show that for an acausal, spacelike, FCC hypersurface $\Sigma$ in a globally hyperbolic spacetime with $\mathcal {C}^{1,1}$-metric the (future) cut locus $\mathrm {Cut}^{+}(\Sigma )\subset M$ has measure zero. This will be vital in the proof of Lemma 3.31.

#### **Definition 3.19**

(*Cut function*) Let $\left( M,g\right)$ with $g\in \mathcal {C}^{1,1}$ be globally hyperbolic and $\Sigma \subset M$ be an acausal, spacelike, FCC hypersurface. The function

$$\begin{aligned} s_{\Sigma }^{+}:S^{+}N\Sigma&\rightarrow \bar{\mathbb {R}}\\ s_{\Sigma }^{+}(v)&:=\sup \left\{ t>0:\,\tau _{\Sigma }(\gamma _{v}(t))=L(\gamma _{v}|_{\left[ 0,t\right] })\right\} \end{aligned}$$

is called the cut function.

We first show measurability of the cut function.

#### **Lemma 3.20**

(Measurability of the cut function) The cut function is measurable with respect to the completion $\mathcal {B}_{\mu _{g}}$ of the Borel-$\sigma$-algebra of $S^{+}N\Sigma$ w.r.t. the measure $\mu _{g}$ induced by the metric *g*.

#### *Proof*

To begin with we rewrite the cut function in a form that makes it possible to use Proposition A.6. Define the set-valued map $F:S^{+}N\Sigma \rightarrow \mathcal {P}(\mathbb {R})$ by

$$\begin{aligned} F(v):=\left\{ t\in \mathbb {R}:\,\left( v,t\right) \in \mathcal {D}\,\mathrm {and}\,\tau _{\Sigma }(\gamma _{v}(t))=L(\gamma _{v}|_{\left[ 0,t\right] })\right\} , \end{aligned}$$

where $\mathcal {D}$ denotes the maximal domain of definition of the flow of the (normal) exponential map. Note that $\mathcal {D}$ is open. Then (using Proposition 3.7)

$$\begin{aligned} s_{\Sigma }^{+}(v)=\sup \left\{ t:\, t\in F(v)\right\} . \end{aligned}$$

Since $\mathbb {R}$ is a Suslin space (see Example A.3) and $f=\mathrm {pr}_{\mathbb {R}}:S^{+}N\Sigma \times \mathbb {R}\rightarrow \mathbb {R}$ is continuous (so in particular measurable) it only remains to show that

$$\begin{aligned} \mathrm {graph}(F)=\left\{ \left( v,t\right) \in \mathcal {D}:\,\tau _{\Sigma }(\gamma _{v}(t))=L(\gamma _{v}|_{\left[ 0,t\right] })\right\} \end{aligned}$$

is measurable. This in turn follows immediately if we can show that both the map $\left( v,t\right) \mapsto \tau _{\Sigma }(\gamma _{v}(t))$ and $(v,t)\mapsto L(\gamma _{v}|_{\left[ 0,t\right] })$ are continuous on $\mathcal {D}$. The first continuity follows from continuity of $\tau _{\Sigma }$ on *M* (see Lemma 3.7) and continuity of $\left( v,t\right) \mapsto \gamma _{v}(t)$ on $\mathcal {D}$ (by continuous dependence of ODE solutions on the initial data). For the second one, note that $\left( v,t\right) \mapsto \int _{0}^{t}g(\gamma _{v}(\tau ))(\dot{\gamma }_{v}(\tau ),\dot{\gamma }_{v}(\tau ))d\tau$ is continuous because the integrand is. $\square$

#### *Remark 3.21*

Note that $\mathcal {B}_{\mu _{g}}$ is actually independent of *g*: If $\tilde{g}$ is any other semi-Riemannian metric on *M* then $\mathcal {B}_{\mu _{g}}=\mathcal {B}_{\mu _{\tilde{g}}}$because the Borel sets of measure zero are the same for $\mu _{g}$ and $\mu _{\tilde{g}}$ as locally any such measure is given by the Lebesque measure multiplied by a positive function (cf. [9, 16.22.2]).

Also, for smooth metrics measurability is a direct consequence of lower semi-continuity of the cut function, but the proof of lower semi-continuity uses the characterization of the cut points as either conjugate points or meeting points of two maximizing geodesics (see, e.g., [3, Prop. 9.7]), which one does not have in the $\mathcal {C}^{1,1}$ case and it is unclear whether lower semi-continuity even remains true for $\mathcal {C}^{1,1}$-metrics.

#### **Definition 3.22**

(*Cut locus*) The tangential (future) cut locus is defined as

$$\begin{aligned} \mathrm {Cut}_{T}^{+}(\Sigma ):=\left\{ s_{\Sigma }^{+}(v)v:\, v\in S^{+}N\Sigma \,\,\mathrm {and}\,\left( v,s_{\Sigma }^{+}(v)\right) \in \mathcal {D}\right\} \subset N\Sigma . \end{aligned}$$

The (future) cut locus is defined as the image of the tangential cut locus under the normal exponential map:

$$\begin{aligned} \mathrm {Cut}^{+}(\Sigma ):=\exp ^{N}(\mathrm {Cut}_{T}^{+}(\Sigma )). \end{aligned}$$

#### **Proposition 3.23**

Let $\left( M,g\right)$ with $g\in \mathcal {C}^{1,1}$ be globally hyperbolic and $\Sigma \subset M$ be an acausal, spacelike, FCC hypersurface. Then the future cut locus $\mathrm {Cut}^{+}(\Sigma )\subset M$ has measure zero.

#### *Proof*

First note that $S^{+}N\Sigma \times (0,\infty )\cong N\Sigma \setminus \left\{ 0\right\}$ via $\left( v,t\right) \mapsto tv$. Using this identification we have

$$\begin{aligned} \mathrm {Cut}_{T}^{+}(\Sigma )=\left\{ \left( v,s_{\Sigma }^{+}(v)\right) :v\in S^{+}N\Sigma \,\,\mathrm {and}\,\left( v,s_{\Sigma }^{+}(v)\right) \in \mathcal {D}\right\} =\mathrm {graph}(s_{\Sigma }^{+})\cap \mathcal {D}. \end{aligned}$$

So from measurability of the cut function (Lemma 3.20) and Proposition A.7 and Proposition A.8 from the appendix, we obtain that the tangential cut locus $\mathrm {Cut}_{T}^{+}(\Sigma )\subset N\Sigma$ has measure zero.

Now the normal exponential map $\exp ^{N}:N\Sigma \rightarrow M$ is a locally Lipschitz continuous map from the $n-1+1=n$-dimensional manifold $N\Sigma$ to the *n*-dimensional manifold *M*, hence its chart representations (with relatively compact domains) can be extended to Lipschitz continuous maps from $\mathbb {R}^{n}\rightarrow \mathbb {R}^{n}$. Using a compact exhaustion $K_{n}$ of $N\Sigma$ and covering each $K_{n}$ by finitely many charts (with relatively compact domains) we see from Proposition A.9 that $\exp ^{N}(K_{n}\cap \mathrm {Cut}_{T}^{+}(\Sigma ))$ has measure zero. Thus $\mathrm {Cut}^{+}(\Sigma )=\bigcup _{n}\exp ^{N}(K_{n}\cap \mathrm {Cut}_{T}^{+}(\Sigma ))$ has measure zero. $\square$

### The comparison manifolds

For any given $\kappa ,\beta$ we use the comparison manifolds $M_{\kappa ,\beta }$ defined in [25, Sec. 4.2]. To make this work more self-contained, we will briefly review their definition and properties.

These comparison manifolds were constructed to satisfy $CCC(\kappa ,\beta )$ with equality in both the Ricci as well as the mean curvature estimates and are given by certain warped products $M_{\kappa ,\beta }=\left( a_{\kappa ,\beta },b_{\kappa ,\beta }\right) \times N_{\kappa ,\beta }$ for $0\in (a_{\kappa ,\beta },b_{\kappa ,\beta })\subset \mathbb {R}$, where $N_{\kappa ,\beta }$ is the $(n-1)$-dimensional simply connected Riemannian manifold with constant sectional curvature of 0, 1, or $-1$, depending on $\kappa$ and $\beta$ (so either $\mathbb {R}^{n-1}$, the unit sphere $S^{n-1}$, or hyperbolic space $H^{n-1}$), with metric

$$\begin{aligned} g_{\kappa ,\beta }=-dt^{2}+f_{\kappa ,\beta }(t)^{2}h, \end{aligned}$$

where *h* denotes the standard Riemannian metric on $N_{\kappa ,\beta }$ and $f_{\kappa ,\beta }:\left( a_{\kappa ,\beta },b_{\kappa ,\beta }\right) \rightarrow \mathbb {R}$ is a positive smooth function.

The warping functions for each pair $\kappa ,\beta$ are summarized in the table below, which is based on [25, Table 1], but we use that the mean curvature $H_{0}$ of the hypersurface $\Sigma _{\kappa ,\beta }:=\{0\}\times N_{\kappa ,\beta }\subset M_{\kappa ,\beta }$ is equal to $\beta$ to express their constant *b* in terms of $\beta$ and we also include the respective constants $b_{\kappa ,\beta }$ that specify the upper bound of the interval where $f_{\kappa ,\beta }^{2}>0$. Also note that our base manifold is assumed to be *n*-dimensional (whereas it is $(n+1)$-dimensional in [25]) and for notational simplicity some of the $f_{\kappa ,\beta }$ listed in Table 1 are strictly negative instead of positive. (Of course one may replace them with $-f_{\kappa ,\beta }$ to obtain positive warping functions).

**Table 1:** Warping functions for different values of $\kappa ,\beta$

$\kappa$	$\beta$	$N_{\kappa ,\beta }$	*b*	$f_{\kappa ,\beta }(t)$	$b_{\kappa ,\beta }$
$\kappa <0$	$\frac{\left| \beta \right| }{(n-1)\sqrt{\left| \kappa \right| }}<1$	$S^{n-1}$	$\tanh ^{-1}(\frac{\beta }{(n-1)\sqrt{\left| \kappa \right| }})$	$\frac{1}{\sqrt{\left| \kappa \right| }}\cosh (\sqrt{\left| \kappa \right| }t+b)$	$\infty$
	$\frac{\left| \beta \right| }{(n-1)\sqrt{\left| \kappa \right| }}=1$	$\mathbb {R}^{n-1}$	0	$\exp (\mathrm {sgn}(\beta )\sqrt{\left| \kappa \right| }t)$	$\infty$
	$\frac{\beta }{(n-1)\sqrt{\left| \kappa \right| }}>1$	$H^{n-1}$	$\coth ^{-1}(\frac{\beta }{(n-1)\sqrt{\left| \kappa \right| }})$	$\frac{1}{\sqrt{\left| \kappa \right| }}\sinh (\sqrt{\left| \kappa \right| }t+b)$	$\infty$
	$\frac{\beta }{(n-1)\sqrt{\left| \kappa \right| }}<-1$	$H^{n-1}$	$\coth ^{-1}(\frac{\beta }{(n-1)\sqrt{\left| \kappa \right| }})$	$\frac{1}{\sqrt{\left| \kappa \right| }}\sinh (\sqrt{\left| \kappa \right| }t+b)$	$-\frac{b}{\sqrt{\left| \kappa \right| }}$
$\kappa =0$	$\beta =0$	$\mathbb {R}^{n-1}$	0	1	$\infty$
	$\beta >0$	$H^{n-1}$	$\frac{n-1}{\beta }$	$t+b$	$\infty$
	$\beta <0$	$H^{n-1}$	$\frac{n-1}{\beta }$	$t+b$	$-\frac{n-1}{\beta }$
$\kappa >0$	$\beta \in \mathbb {R}\setminus \{0\}$	$H^{n-1}$	$\cot ^{-1}(\frac{\beta }{(n-1)\sqrt{\kappa }})$	$\frac{1}{\sqrt{\kappa }}\sin (\sqrt{\kappa }t+b)$	$\frac{-b+\frac{\pi }{2}(1+\mathrm {sgn}(\beta ))}{\sqrt{\kappa }}$
	$\beta =0$	$H^{n-1}$	$\frac{\pi }{2}$	$\frac{1}{\sqrt{\kappa }}\cos (\sqrt{\kappa }t)$	$\frac{\pi }{2\sqrt{\kappa }}$

Since $\lim _{t\nearrow b_{\kappa ,\beta }}f_{\kappa ,\beta }(t)=0$ if $b_{\kappa ,\beta }<\infty$ we may define continuous functions $\tilde{f}_{\kappa ,\beta }:[0,\infty )\rightarrow \mathbb {R}$ by extending $f_{\kappa ,\beta }$ by zero if necessary, so

15 $$\begin{aligned} \tilde{f}_{\kappa ,\beta }(t):= {\left\{ \begin{array}{ll} f_{\kappa ,\beta }(t) &{} t<b_{\kappa ,\beta }\\ 0 &{} t\ge b_{\kappa ,\beta }. \end{array}\right. } \end{aligned}$$

We now investigate some of the circumstances under which convergence of $\kappa \nearrow \kappa _{0}$ and $\beta \searrow \beta _{0}$ (since the approximating metrics we will use satisfy Lemma 3.13 and Lemma 3.15) implies pointwise convergence of the corresponding $\tilde{f}_{\kappa ,\beta }$ or at least of the functions $t\mapsto \frac{\tilde{f}_{\kappa ,\beta }(t)}{f_{\kappa ,\beta }(0)}$. Clearly the map $\left( \kappa ,\beta \right) \mapsto \tilde{f}_{\kappa ,\beta }$ is continuous (w.r.t. pointwise convergence) on $\left( \mathbb {R}_{>0}\times \mathbb {R}\setminus \{0\}\right) \cup \{(\kappa ,\beta )\in \mathbb {R}^{2}:\,\kappa <0\,\mathrm {and}\,\left| \beta \right| \ne (n-1)\sqrt{|\kappa |}\}$. For the remaining points, simple calculations show the following:

#### **Lemma 3.24**

If either

1. $$\kappa _{0}=0$$, $$\beta _{0}\ne 0$$ and $$\kappa \nearrow 0$$ and $$\beta \rightarrow \beta _{0}$$,
2. $\kappa _{0}=0$, $\beta _{0}=0$ and $\kappa \nearrow 0$ and $\beta :=(n-1)\sqrt{|\kappa |}\searrow 0$, or
3. $$\kappa _{0}>0$$, $$\beta _{0}=0$$ and $$\kappa \rightarrow \kappa _{0}$$ and $$\beta \searrow 0$$,
4. $$\kappa _{0}<0,$$ $$\beta _{0}=(n-1)\sqrt{|\kappa _{0}|}$$ and $$\kappa \nearrow \kappa _{0}$$ and $$\beta :=(n-1)\sqrt{|\kappa |}\searrow \beta _{0}$$

then $\tilde{f}_{\kappa ,\beta }\rightarrow \tilde{f}_{\kappa _{0},\beta _{0}}$ pointwise. And if

$$\begin{aligned} \kappa _{0}<0,\,\beta _{0}=-(n-1)\sqrt{|\kappa _{0}|}\,\mathrm {and}\,\kappa \nearrow \kappa _{0}\,\mathrm {and}\,\beta \searrow \beta _{0} \end{aligned}$$

then $\tilde{f}_{\kappa ,\beta }\not \rightarrow \tilde{f}_{\kappa _{0},\beta _{0}}$ but still $\frac{\tilde{f}_{\kappa ,\beta }(t)}{f_{\kappa ,\beta }(0)}\rightarrow \frac{\tilde{f}_{\kappa _{0},\beta _{0}}(t)}{f_{\kappa _{0},\beta _{0}}(0)}$ for all $t\ge 0$.

Furthermore, for any $\kappa \le 0$ and $\beta \in \mathbb {R}$ one has $\left| \frac{\tilde{f}_{\kappa ,\beta }(t)}{f_{\kappa ,\beta }(0)}\right| \le \max \left\{ \left| \frac{\tilde{f}_{\kappa ,\beta }(T)}{f_{\kappa ,\beta }(0)}\right| ,1\right\}$ for all $t\le T$ because $\left| \tilde{f}_{\kappa ,\beta }\right|$ is monotone or convex and for $\kappa >0$ and $\beta \in \mathbb {R}$ we have $\left| \tilde{f}_{\kappa ,\beta }(t)\right| \le \frac{1}{\sqrt{\kappa }}$ for all $t\in \mathbb {R}$.

This convergence result can be used to show convergence of areas and volumes of future spheres and balls in $M_{\kappa ,\beta }$ above a subset of $\Sigma _{\kappa ,\beta }=\{0\}\times N_{\kappa ,\beta }\subset M_{\kappa ,\beta }$ (which is an acausal, spacelike, FCC hypersurface in $M_{\kappa ,\beta }$).

#### **Definition 3.25**

(Future spheres and balls) For any $t>0$ and $A\subset \Sigma$ we define the spheres $S_{A}^{+}(t)$ and balls $B_{A}^{+}(t)$ of time *t* above *A* by

$$\begin{aligned} S_{A}^{+}(t):&=\{p\in I^{+}(\Sigma ):\,\exists q\in A\,\mathrm {with}\, d(q,p)=\tau _{\Sigma }(p)=t\}\,\mathrm {and}\\ B_{A}^{+}(t):&=\bigcup _{s\in \left( 0,t\right) }S_{A}^{+}(s) \end{aligned}$$

Using these definitions and Lemma 3.24 we show:

#### **Corollary 3.26**

For any $\left( \kappa ,\beta \right) \in \mathbb {R}^{2}$ there exist sequences $\delta _{n}>0$ and $\eta _{n}>0$ converging to zero such that

16 $$\begin{aligned} \frac{\mathrm {area}_{\kappa -\delta _{n},\beta +\eta _{n}}S_{B_{n}}^{+}(t)}{\mathrm {area}_{\kappa -\delta _{n},\beta +\eta _{n}}B_{n}}\rightarrow \frac{\mathrm {area}_{\kappa ,\beta }S_{B}^{+}(t)}{\mathrm {area}_{\kappa ,\beta }B} \end{aligned}$$

and

17 $$\begin{aligned} \frac{\mathrm {vol}_{\kappa -\delta _{n},\beta +\eta _{n}}B_{B_{n}}^{+}(t)}{\mathrm {area}_{\kappa -\delta _{n},\beta +\eta _{n}}B_{n}}\rightarrow \frac{\mathrm {vol}_{\kappa ,\beta }B_{B}^{+}(t)}{\mathrm {area}_{\kappa ,\beta }B} \end{aligned}$$

for all $t>0$ and any measurable $B_{n}\subset \Sigma _{\kappa -\delta _{n},\beta +\eta _{n}}$ and measurable $B\subset \Sigma _{\kappa ,\beta }$.

#### *Proof*

The first statement is an immediate consequence of the previous lemma and $\frac{\mathrm {area}_{\kappa ,\beta }S_{B}^{+}(t)}{\mathrm {area}_{\kappa ,\beta }B}=(\frac{\tilde{f}_{\kappa ,\beta }(t)}{f_{\kappa ,\beta }(0)})^{n-1}$ for any $\kappa ,\beta$ (see [25, eq. (15)] and note that $S_{B}^{+}(t)=\emptyset$ for $t\ge b_{\kappa ,\beta }$). For (17) note that and $\mathrm {vol}_{\kappa ,\beta }B_{B}^{+}(t)=\int _{0}^{t}\mathrm {area}_{\kappa ,\beta }S_{B}^{+}(\tau )d\tau$ and that we may apply dominated convergence since for $\kappa \le 0$ and $\beta \in \mathbb {R}$ one has

$$\begin{aligned} \left| \frac{\tilde{f}_{\kappa -\delta _{n},\beta +\eta _{n}}(\tau )}{f_{\kappa -\delta _{n},\beta +\eta _{n}}(0)}\right| \le \max \left\{ \left| \frac{\tilde{f}_{\kappa -\delta _{n},\beta +\eta _{n}}(T)}{f_{\kappa -\delta _{n},\beta +\eta _{n}}(0)}\right| ,1\right\} \rightarrow \max \left\{ \left| \frac{\tilde{f}_{\kappa ,\beta }(T)}{f_{\kappa ,\beta }(0)}\right| ,1\right\} \end{aligned}$$

for all $\tau \le t$ and for $\kappa >0,\beta \in \mathbb {R}$ one has $\left| \frac{\tilde{f}_{\kappa -\delta _{n},\beta +\eta _{n}}(\tau )}{f_{\kappa -\delta _{n},\beta +\eta _{n}}(0)}\right| \le \frac{(\kappa -\delta _n)^{-\frac{1}{2}}}{\left| f_{\kappa -\delta _{n},\beta +\eta _{n}}(0)\right| }\rightarrow \frac{1}{\sqrt{\kappa }\left| f_{\kappa ,\beta }(0)\right| }$.      $\square$

#### *Remark 3.27*

The reason we only show this for specific sequences $\delta _{n}$ and $\eta _{n}$ lies in our somewhat incomplete treatment of the dependence of ${\tilde{f}_{\kappa ,\beta }}/{\tilde{f}_{\kappa ,\beta }(0)}$ on $\kappa ,\beta$ in Lemma 3.24: While it does seem reasonable that the result remains true for all such sequences, that would require many additional cases of possible convergence to be checked in Lemma 3.24, which is rather tedious and completely unnecessary for the rest of this work.

### Volume comparison

We first need to show area and volume comparison statements for the approximating metrics and to do so we need to define future spheres that avoid the cut locus.

#### **Definition 3.28**

For $$t>0$$ let

$$\begin{aligned} \mathcal {S}_{A}^{+}(t):=S_{A}^{+}(t)\setminus \mathrm {Cut}^{+}(\Sigma ). \end{aligned}$$

Similarly, but using the approximations $g_{\varepsilon }$ (from Sect. 3.2), the $g_{\varepsilon }$-time separation $\tau _{\varepsilon ,\Sigma }$ and the $\varepsilon$-cut locus, we define $\mathcal {S}_{\varepsilon ,A}^{+}(t)$.

Using results from [25] we are now able to prove area and volume comparison statements for the approximating metrics.

#### **Proposition 3.29**

(Area comparison for approximations) Let $\kappa ,\beta \in \mathbb {R}$, $g\in \mathcal {C}^{1,1}$ and assume $\left( M,g,\Sigma \right)$ is globally hyperbolic and satisfies $CCC(\kappa ,\beta )$. Let $A\subset \Sigma$ be compact, $\eta ,\delta >0$, $B\subset \Sigma _{\kappa -\delta ,\beta +\eta }$ (with finite, non-zero area) and $T>0$ such that all timelike, f.d. unit speed *g*-geodesics starting in *A* orthogonally to $\Sigma$ exist until at least *T*. Then there exists $\varepsilon _{0}>0$ (depending on $\eta ,\delta ,A,T$) such that for all $\varepsilon <\varepsilon _{0}$ the function

$$\begin{aligned} t\mapsto \frac{\mathrm {area}_{\varepsilon }\,\mathcal {S}_{\varepsilon ,A}^{+}(t)}{\mathrm {area}_{\kappa -\delta ,\beta +\eta }S_{B}^{+}(t)}, \end{aligned}$$

is nonincreasing on (0, *T*] if $T<b_{\kappa -\delta ,\beta +\eta }$ or on $(0,b_{\kappa -\delta ,\beta +\eta })$ if $T\ge b_{\kappa -\delta ,\beta +\eta }$.

#### *Proof*

We would like to use [25, Thm. 8], however, we have to argue that the bounds on Ricci and mean curvature from Lemma 3.13 and 3.15 are sufficient to show this for smooth metrics ([25] requires global bounds while we only have them on compact subsets of *TM* respectively $\Sigma$).

First we note that by compactness of $S^{+}NA$ there exists a neighborhood *U* of $S^{+}NA$ in *TM* such that all *g*-geodesics starting in *U* exist until at least *T*. Then by Lemma 3.18, for $\varepsilon _{0}$ small the set $K_{\varepsilon _{0}}:=\bigcup _{0\le \varepsilon \le \varepsilon _{0}}S_{\varepsilon }^{+}N_{\varepsilon }A\subset TM$ is compact and contained in *U*, hence any *g*-geodesic starting in it exists until *T*. So by Prop. 3.16 there exists $\varepsilon _{0}>\tilde{\varepsilon }_{0}>0$ such that $f\left( \left[ 0,\tilde{\varepsilon }_{0}\right] \times \left[ 0,T\right] \times K_{\varepsilon _{0}}\right) =:\tilde{K}\subset TM$ is compact, in particular *h*-norm bounded by a constant *C*.

From here the proof proceeds analogously to [25, Thm. 8]. Let $0<t_{1}<t_{2}<\min (T,b_{\kappa -\delta ,\beta +\eta })$ and choose a sequence of compact sets $K_{\varepsilon ,j}\subset \mathcal {S}_{\varepsilon ,A}^{+}(t_{2})$ with $\mathrm {area}\, K_{\varepsilon ,j}\nearrow \mathrm {area}\,\mathcal {S}_{\varepsilon ,A}^{+}(t_{2})$. Now, as in [25], we get sets

$$\begin{aligned} K_{\varepsilon ,j}(t):=\Phi _{\varepsilon ,t-t_{2}}(K_{\varepsilon ,j})\subset \mathcal {S}_{\varepsilon ,K_{i}}^{+}(t), \end{aligned}$$

where $\Phi$ is the flow of $-\mathrm {grad}(\tau _{\varepsilon ,\Sigma })$, and

$$\begin{aligned} \frac{d}{dt}\log (\mathrm {area}\, K_{\varepsilon ,j}(t))=\frac{1}{\mathrm {area}\, K_{\varepsilon ,j}(t)}\int _{K_{\varepsilon ,j}(t)}H_{\varepsilon ,t}(q)d\mu _{\varepsilon ,t}(q). \end{aligned}$$

Next we show that for $\varepsilon$ small enough (depending on $\eta$, $\delta$, *A* and $\tilde{K}$)

18 $$\begin{aligned} H_{\varepsilon ,t}(q)\le H_{\kappa -\delta ,\beta +\eta }(t) \end{aligned}$$

for all $q\in K_{\varepsilon ,j}(t)$. This proceeds similarly to [25, Thm. 7]: For any $q\in \mathcal {S}_{\varepsilon ,K_{j}}^{+}(t)$ the unique, maximizing, unit-speed $g_{\varepsilon }$-geodesic $\gamma _{\varepsilon }$ connecting *q* to $\Sigma$ satisfies $\dot{\gamma }_{\varepsilon }(0)\in S_{\varepsilon }^{+}N_{\varepsilon }A$ and we have $H(\gamma (0))\le \beta +\eta$ (by Lemma 3.15) and $\dot{\gamma }_{\varepsilon }\subset \tilde{K}$ and hence $\mathbf {Ric}_{\varepsilon }(\dot{\gamma }_{\varepsilon }(s),\dot{\gamma }_{\varepsilon }(s))\ge n\,(\kappa -\delta )$ for all $s\in [0,t]$ (by Lemma 3.13). Note that this is all that is needed to apply the Riccati comparison argument used in the proof of Thm. 7 and it is the only place where the curvature estimates enter the proof. So we get (18). The remainder of the proof is completely analogous to [25, Thm. 8]. $\square$

#### **Proposition 3.30**

(Volume comparison for approximations) Let $\kappa ,\beta \in \mathbb {R}$, $g\in \mathcal {C}^{1,1}$ and assume $\left( M,g,\Sigma \right)$ is globally hyperbolic and satisfies $CCC(\kappa ,\beta )$. Let $A\subset \Sigma$ be compact, $\eta ,\delta >0$, $B\subset \Sigma _{\kappa -\delta ,\beta +\eta }$ (with finite, non-zero area) and $T>0$ such that all timelike, f.d. unit speed geodesics starting orthogonally to *A* exist until at least *T*. Then there exists $\varepsilon _{0}>0$ (depending on $\eta ,\delta ,A,T$) such that for all $\varepsilon <\varepsilon _{0}$ the function

$$\begin{aligned} t\mapsto \frac{\mathrm {vol}_{\varepsilon }\, B_{\varepsilon ,A}^{+}(t)}{\mathrm {vol}_{\kappa -\delta ,\beta +\eta }B_{B}^{+}(t)} \end{aligned}$$

is nonincreasing on (0, *T*] if $T<b_{\kappa -\delta ,\beta +\eta }$ and on $(0,\infty )$ if $T\ge b_{\kappa -\delta ,\beta +\eta }$.

#### *Proof*

For $T<b_{\kappa -\delta ,\beta +\eta }$ this follows from the area comparison result by using the coarea formula (see [25, Thm. 9] for details). Since $b_{\kappa -\delta ,\beta +\eta }$ is defined by being the upper bound of the maximal interval of positivity of the warping function $f_{\kappa -\delta ,\beta +\eta }$, we either have $b_{\kappa -\delta ,\beta +\eta }=\infty$ or $b_{\kappa -\delta ,\beta +\eta }<\infty$ and $\lim _{t\nearrow b_{\kappa -\delta ,\beta +\eta }}f_{\kappa -\delta ,\beta +\eta }(t)=0$. In the second case an argument completely analogous to the area comparison proof of [25, Thm. 10] shows that $S_{\varepsilon ,\Sigma }^{+}(t)=\emptyset$ for $t>b_{\kappa -\delta ,\beta +\eta }$. Hence by the coarea formula (see [25, Prop. 3] ) $t\mapsto \text {vol}_\varepsilon (B_{\varepsilon ,A}^{+}(t))$ remains constant for $t>b_{\kappa -\delta ,\beta +\eta }$ and thus $\frac{\mathrm {vol}_{\varepsilon }\, B_{\varepsilon ,A}^{+}(t)}{\mathrm {vol}_{\kappa -\delta ,\beta +\eta }B_{B}^{+}(t)}$ remains nonincreasing for $t>b_{\kappa -\delta ,\beta +\eta }$. $\square$

The plan is now to use Proposition 3.30 and first let $\varepsilon \rightarrow 0$ and then $\delta ,\eta \rightarrow 0$. To make the proof more readable, we first show that $\mathrm {vol}_{\varepsilon }\, B_{\varepsilon ,A}^{+}(t)\rightarrow \mathrm {vol}\, B_{A}^{+}(t)$ in a separate lemma.

#### **Lemma 3.31**

(Volume convergence) Let $g\in \mathcal {C}^{1,1}$, assume (*M*, *g*) is globally hyperbolic and let $\Sigma \subset M$ be an acausal, spacelike, FCC hypersurface. Let $A\subset \Sigma$ be compact with $\mu _{\Sigma }(\partial A)=0$ (where $\partial A$ is the boundary of *A* in $\Sigma$) and $T>0$ such that all timelike, f.d., unit-speed geodesics starting orthogonally to *A* exist until at least *T*. Then for any $0<t\le T$ we have

$$\begin{aligned} \mathrm {vol}_{\varepsilon }\, B_{\varepsilon ,A}^{+}(t)\rightarrow \mathrm {vol}\, B_{A}^{+}(t) \end{aligned}$$

for $$\varepsilon \rightarrow 0$$.

#### *Proof*

From Proposition 3.16 and Lemma 3.18 it follows in a similar way as in the beginning of the proof of Proposition 3.29 that $\bigcup _{0\le \varepsilon \le \varepsilon _{0}}\bar{B}_{\varepsilon ,A}^{+}(T)$ (where $\bar{B}_{A}^{+}(t):=\{p\in I^{+}(\Sigma ):\exists q\in A\, s.t.\,\tau _{\Sigma }(p)=d(p,q)\le t\}$ for $t>0$) is contained in the compact set $K=f([0,\varepsilon _{0}]\times [0,T]\times K_{\varepsilon _{0}})$ (with *f* as in Proposition 3.16). Now fix $0<t\le T$ then $B_{\varepsilon ,A}^{+}(t)\subset \bar{B}_{\varepsilon ,A}^{+}(T)\subset K$ for all $0\le \varepsilon \le \varepsilon _{0}$ and $B_{\varepsilon ,A}^{+}(t)\subset I_{\varepsilon }^{+}(\Sigma )\subset I^{+}(\Sigma )$ (note that we chose $g_{\varepsilon }$ such that $g_{\varepsilon }\prec g$). So it only remains to show that the functions $\chi _{B_{\varepsilon ,A}^{+}(t)}\,\sqrt{\left| \det g_{\varepsilon ,ij}\right| }\rightarrow \chi _{B_{A}^{+}(t)}\,\sqrt{\left| \det g_{ij}\right| }$ pointwise almost everywhere on $K\cap I^{+}(\Sigma )$ and then apply dominated convergence. This is clear for $\sqrt{\left| \det g_{\varepsilon ,ij}\right| }$, so we only have to look at the characteristic functions.

First note that $\mu (\mathrm {Cut}^{+}(\Sigma ))=0$ by Proposition 3.23, so it suffices to show convergence a.e. on $(K\cap I^{+}(\Sigma ))\setminus \mathrm {Cut}^{+}(\Sigma )$. For any $p\in (K\cap I^{+}(\Sigma ))\setminus \mathrm {Cut}^{+}(\Sigma )$ there exists a *unique* (up to reparametrization) causal curve $\gamma ^{p}$ maximizing the distance from *p* to $\Sigma$ (and this curve is a geodesic starting orthogonally to $\Sigma$): Existence follows from Lemma 3.7 and if there were two different maximizing geodesics none of them could be maximizing past *p* (since locally any maximizing timelike curve has to be an unbroken geodesic, see [18, Thm. 6]) and hence $p\in \mathrm {Cut}^{+}(\Sigma )$ by the definition of the cut locus. This allows us to split $(K\cap I^{+}(\Sigma ))\setminus \mathrm {Cut}^{+}(\Sigma )$ into five (not necessarily mutually distinct) subsets:

1. we have $L\left( \gamma ^{p}\right) <t$ and $\gamma ^{p}(0)\in A^{\circ }$ (where $A^{\circ }$ is the interior of *A* as a subset of $\Sigma$), i.e. $p\in B_{A^{\circ }}^{+}(t)$, or
2. $L\left( \gamma ^{p}\right)$ arbitrary and $\gamma ^{p}(0)\notin A$, in particular $p\notin \bar{B}_{A}^{+}(t)$, or
3. $L\left( \gamma ^{p}\right) >t$ and $\gamma ^{p}(0)$ arbitrary, so again $p\notin \bar{B}_{A}^{+}(t)$, or
4. $$L\left( \gamma ^{p}\right) =t$$ and $$\gamma ^{p}(0)\in A$$, i.e. $$p\in S_{A}^{+}(t)$$, or
5. $$L\left( \gamma ^{p}\right) \le t$$ and $$\gamma ^{p}(0)\in \partial A=A\setminus A^{\circ }$$, i.e. $$p\in \bar{B}_{\partial A}^{+}(t)$$

We now show that in cases $\left( 1\right) -\left( 3\right)$ the characteristic functions converge in *p*:

In case $\left( 3\right)$ we have $L_{g}(\gamma ^{p})>t$. But then for $\varepsilon$ small this $\gamma ^{p}$ is also $g_{\varepsilon }$ timelike and Lemma 4.2 from [14] gives that for any small $\delta >0$ there exists $\varepsilon _0$ such that for all $\varepsilon \le \varepsilon _0$

19 $$\begin{aligned} \tau _{\varepsilon ,\Sigma }(p)\ge L_{\varepsilon }(\gamma ^{p})>L_{g}(\gamma ^{p})-\delta >t. \end{aligned}$$

Thus $p\notin \bar{B}_{\varepsilon ,A}^{+}(t)$ for $\varepsilon$ small.

Now for case $\left( 1\right)$, let $p\in B_{A^{\circ }}^{+}(t)\subset B_{A}^{+}(t)$. Let $\gamma _{\varepsilon }$ be a $g_{\varepsilon }$-geodesic between $q_{\varepsilon }\in \Sigma$ and *p* with $L_{\varepsilon }(\gamma _{\varepsilon })=\tau _{\varepsilon ,\Sigma }(p)$. From $q_{\varepsilon }\in J^{-}(p)\cap \Sigma$, it follows that $\gamma _{\varepsilon }\subset J^{-}(p)\cap J^{+}(J^{-}(p)\cap \Sigma )$ for all $\varepsilon$, which is compact by Remark 3.5. This allows us to use Lemma 4.2 from [14] to obtain that for any small $\delta >0$ there exists $\varepsilon _{0}$ such that

20 $$\begin{aligned} \tau _{\varepsilon ,\Sigma }(p)=L_{\varepsilon }(\gamma _{\varepsilon })<L_{g}(\gamma _{\varepsilon })+\delta \le \tau _{\Sigma }(p)+\delta . \end{aligned}$$

for all $\varepsilon \le \varepsilon _{0}$. This shows that if $\tau _{\Sigma }(p)<t$, then $\tau _{\varepsilon ,\Sigma }(p)<t$ for small $\varepsilon$. Now let $U\subset A^{\circ }$ be a neighborhood of $\gamma ^{p}(0)$ in $\Sigma$. It remains to show that $q_{\varepsilon }\in U\subset A^{\circ }$ for small $\varepsilon$. Assume the contrary and let $\gamma _{\varepsilon _{j}}$ be a subsequence with $q_{\varepsilon _{j}}\notin U$. By our limit curve Lemma 3.6, we may assume (after reparametrizing and passing to a further subsequence) that $\gamma _{\varepsilon _{j}}$ converges to a causal curve $\tilde{\gamma }$ from $q:=\tilde{\gamma }(0)=\lim q_{\varepsilon _{j}}\notin U$ to *p* with $L_{g}(\tilde{\gamma })\ge \limsup _{j\rightarrow \infty }L_{g}(\gamma _{\varepsilon _{j}})$. Using (20) and (19) gives

$$\begin{aligned} L_{g}(\tilde{\gamma })\ge \limsup _{j\rightarrow \infty }L_{g}(\gamma _{\varepsilon _{j}})\ge \limsup _{j\rightarrow \infty }\tau _{\varepsilon _{j},\Sigma }(p)-\delta \ge L_{g}(\gamma ^{p})-2\delta =\tau _{\Sigma }(p)-2\delta \end{aligned}$$

for any $\delta >0$ and letting $\delta \rightarrow 0$ shows that $\tilde{\gamma }$ is also maximizing the distance between *p* and $\Sigma$, giving a contradiction, since $\tilde{\gamma }(0)\ne \gamma ^{p}(0)$ but $\gamma ^{p}$ is the unique causal curve realizing the distance from $\Sigma$ to *p* by definition. Altogether, $p\in B_{\varepsilon ,A^{\circ }}^{+}(t)\subset B_{\varepsilon ,A}^{+}(t)$ for small enough $\varepsilon$.

Next we look at case $\left( 2\right)$, i.e., $\gamma ^{p}(0)\notin A$ (and thus $p\notin B_{A}^{+}(t)$). Let *U* be a neighborhood of $\gamma ^{p}(0)$ in $\Sigma$ with $U\cap A=\emptyset$ (this exists since *A* is closed). By the argument presented when dealing with case $\left( 1\right)$, we have that for $\varepsilon$ small enough $\gamma _{\varepsilon }(0)\in U$, hence not in *A* and so $p\notin B_{\varepsilon ,A}^{+}(t)$.

It remains deal with cases $\left( 4\right)$ and $\left( 5\right)$. Here we show that both $S_{A}^{+}(t)$ and $\bar{B}_{\partial A}^{+}(t)$ are contained in sets of measure zero.

Regarding $S_{A}^{+}(t)$, let $\tilde{\mathbf {n}}$ be a $\mathcal {C}^{1,1}$-extension of $\mathbf {n}$ to some small neighborhood *U* of *A* (in *M*) and consider the map $h:\, p\mapsto \exp _{p}(t\tilde{\mathbf {n}}(p))$. For *U* small enough this is well defined on *U* (by a standard ODE argument) and because the exponential map is locally Lipschitz continuous, this map is as well. Now since $S_{A}^{+}(t)\subset h(A)$, $\mu (A)=0$ (because $A\subset \Sigma$), *A* is compact and any Lipschitz map from $\mathbb {R}^{n}\rightarrow \mathbb {R}^{n}$ maps sets of (Lebesgue-)measure zero to sets of measure zero (see Proposition A.9 in the appendix), we have that *h*(*A*) has measure zero.

Finally, for $\bar{B}_{\partial A}^{+}(t)$, note that $\partial A\subset A$ and hence all f.d., unit-speed, normal geodesics starting in $\partial A$ exist until at least $T\ge t$, so $\partial A\times [0,t]\subset \mathcal {D}$ and $\bar{B}_{\partial A}^{+}(t)\subset \exp ^{N}\left( [0,t]\cdot \partial A\right)$. Now since $[0,t]\cdot \partial A\subset N\Sigma$ has measure zero (because by assumption $\mu _{\Sigma }(\partial A)=0$) and is compact (by compactness of *A*) and $\exp ^{N}$ is locally Lipschitz, the desired result follows again from Prop. A.9.

Altogether, this shows that indeed $\chi _{B_{\varepsilon ,A}^{+}(t)}\rightarrow \chi _{B_{A}^{+}(t)}\,$ pointwise almost everywhere. $\square$

We are now ready to prove Theorem 1.1.

#### *Proof of Theorem 1.1*

Let $0<t_{1}<t_{2}\le T$. It suffices to show that

$$\begin{aligned} \mathrm {vol}\, B_{A}^{+}(t_{2})\le \mathrm {vol}\, B_{A}^{+}(t_{1})\,\frac{\mathrm {vol}_{\kappa ,\beta }B_{B}^{+}(t_{2})}{\mathrm {vol}_{\kappa ,\beta }B_{B}^{+}(t_{1})}. \end{aligned}$$

By Lemma 3.31 we have

$$\begin{aligned} \mathrm {vol}_{\varepsilon }\, B_{\varepsilon ,A}^{+}(t)\rightarrow \mathrm {vol}\, B_{A}^{+}(t) \end{aligned}$$

for all $t\in (0,T]$. So using Proposition 3.30 and letting $\varepsilon \rightarrow 0$ shows that for all $\eta ,\delta >0$ and $B_{\delta ,\eta }\subset \Sigma _{\kappa -\delta ,\beta +\eta }$ (with $0<\mathrm {area}_{\kappa -\delta ,\beta +\eta }B_{\delta ,\eta }<\infty$)

$$\begin{aligned} \mathrm {vol}\, B_{A}^{+}(t_{2})\le \mathrm {vol}\, B_{A}^{+}(t_{1})\,\frac{\mathrm {vol}_{\kappa -\delta ,\beta +\eta }B_{B_{\delta ,\eta }}^{+}(t_{2})}{\mathrm {vol}_{\kappa -\delta ,\beta +\eta }B_{B_{\delta ,\eta }}^{+}(t_{1})} \end{aligned}$$

Now by (17) there exist sequences $\delta _{n},\eta _{n}\rightarrow 0$ such that

$$\begin{aligned} \mathrm {vol}_{\kappa -\delta _{n},\beta +\eta _{n}}B_{B_{n}}^{+}(t)\rightarrow \mathrm {vol}_{\kappa ,\beta }B_{B}^{+}(t)\,\frac{\mathrm {area}_{\kappa -\delta _{n},\beta +\eta _{n}}B_{n}}{\mathrm {area}_{\kappa ,\beta }B}, \end{aligned}$$

for all $t>0$ which implies

$$\begin{aligned} \frac{\mathrm {vol}_{\kappa -\delta _{n},\beta +\eta _{n}}B_{B_{n}}^{+}(t_{2})}{\mathrm {vol}_{\kappa -\delta _{n},\beta +\eta _{n}}B_{B_{n}}^{+}(t_{1})}\rightarrow \frac{\mathrm {vol}_{\kappa ,\beta }B_{B}^{+}(t_{2})}{\mathrm {vol}_{\kappa ,\beta }B_{B}^{+}(t_{1})}. \end{aligned}$$

So $t\mapsto \frac{\mathrm {vol}\, B_{A}^{+}(t)}{\mathrm {vol}_{\kappa ,\beta }B_{B}^{+}(t)}$ is indeed nonincreasing on (0, *T*]. $\square$

## Applications

### Myers’ theorem for $\mathcal {C}^{1,1}$-metrics

We will use the volume comparison result Theorem 2.4 to give a proof of Myers’ theorem for $\mathcal {C}^{1,1}$-metrics.

#### **Theorem 4.1**

Let $\left( M,g\right)$ be a complete *n*-dimensional Riemannian manifold with $\mathcal {C}^{1,1}$-metric *g* such that $\mathbf {Ric}\ge \left( n-1\right) \kappa \, g$ for some $\kappa >0$. Then $\text {diam}\left( M\right) \le \frac{\pi }{\sqrt{\kappa }}$.

#### *Proof*

Let $S_{\kappa }^{n}$ be the *n*-dimensional sphere of radius $\kappa$ with the standard metric, then $S_{\kappa }^{n}$ has constant sectional curvature $\kappa$ and $\text {diam}\left( S_{\kappa }^{n}\right) =\frac{\pi }{\sqrt{\kappa }}$. Clearly $\mathrm {vol}_{\kappa }B^{\kappa }(r)$ is constant in *r* for $r\ge \frac{\pi }{\sqrt{\kappa }}$. By Theorem 2.4 this implies that also

$$\begin{aligned} r\mapsto \mathrm {vol}B_{p}(r) \end{aligned}$$

is constant for all $r\ge \frac{\pi }{\sqrt{\kappa }}$ and all $p\in M$. Fix *p* and assume there exists $q\in M$ with $d(p,q)>\frac{\pi }{\sqrt{k}}$. Then by continuity of *d*(., *p*) we find a neighborhood *U* of *q* with $\mu (U)\ne 0$ such that $d(p,q)+1>d(x,p)>\frac{\pi }{\sqrt{\kappa }}$ for all $x\in U$, so $U\subset B_{p}(d(p,q)+1)$ and $U\cap B_{p}(\frac{\pi }{\sqrt{\kappa }})=\emptyset$. But this shows that

$$\begin{aligned} \mathrm {vol}B_{p}(d(p,q)+1)>\mathrm {vol}B_{p}\left( \frac{\pi }{\sqrt{\kappa }}\right) , \end{aligned}$$

contradicting $r\mapsto \mathrm {vol}B_{p}(r)$ being constant for all $r\ge \frac{\pi }{\sqrt{\kappa }}$. $\square$

This result is not very surprising since it is known that there are generalizations of Myers’ theorem even for metric measure spaces (see Cor. 2.6 in [24]). However, these do not immediately imply Theorem 4.1 above, because for metric measure spaces the needed curvature bound is (by necessity) defined in a different manner from $\mathbf {Ric}\ge \left( n-1\right) \kappa \, g$ in $L_{\mathrm {loc}}^{\infty }$.

### Hawking’s singularity theorem for $\mathcal {C}^{1,1}$-metrics

We first show a general result concerning geodesic incompleteness of globally hyperbolic manifolds.

#### **Theorem 4.2**

Assume that $\left( M,g,\Sigma \right)$ (with $g\in \mathcal {C}^{1,1}$) is globally hyperbolic and satisfies the $CCC(\kappa ,\beta )$ condition with either

1. $$\kappa >0$$, $$\beta \in \mathbb {R}$$,
2. $$\kappa =0$$, $$\beta <0$$ or
3. $$\kappa <0$$, $$\beta <0$$ such that $$\frac{\beta }{(n-1)\,\sqrt{\left| \kappa \right| }}<-1$$.

Then $\tau _{\Sigma }(p)\le b_{\kappa ,\beta }<\infty$ for all $p\in I^{+}(\Sigma )$ and $\left( M,g\right)$ is timelike future geodesically incomplete.

#### *Proof*

First note that for these values of $\kappa$ and $\beta$ we have $b_{\kappa ,\beta }<\infty$ (see Table 1), so $\tau _{\Sigma }(p)\le b_{\kappa ,\beta }$ for all $p\in I^{+}(\Sigma )$ implies $L\left( \gamma \right) \le b_{\kappa ,\beta }$ for all timelike, f.d. geodesics $\gamma$ starting in $\Sigma$, which implies incompleteness of *M*.

Now assume to the contrary that there exists $p\in I^{+}(\Sigma )$ with $\tau _{\Sigma }(p)>b_{\kappa ,\beta }$. We first argue that we may w.l.o.g. assume $p\notin \mathrm {Cut}^{+}(\Sigma )$: By continuity of $\tau _{\Sigma }$ (see Lemma 3.7) there is a neighborhood *U* of *p* such that $\tau _{\Sigma }(q)>b_{\kappa ,\beta }$ for all $q\in U$ and since $\mathrm {Cut}^{+}(\Sigma )$ has measure zero (see 3.23) but *U* does not there exists $\tilde{p}\notin \mathrm {Cut}^{+}(\Sigma )$ with $\tau _{\Sigma }(\tilde{p})>b_{\kappa ,\beta }$.

Now, if we have $p\notin \mathrm {Cut}^{+}(\Sigma )$ then, by the same argument as in the proof of Lemma 3.31, there exists a *unique* unit-speed geodesic $\gamma ^{p}$ from $\gamma ^{p}(0)\in \Sigma$ to *p* with $L\left( \gamma ^{p}\right) =\tau _{\Sigma }(p)>b_{\kappa ,\beta }$ (and this geodesic has to start orthogonally to $\Sigma$ by Lemma 3.7). In particular, $\gamma ^{p}$ exists until at least some $T>\tau _{\Sigma }(p)>b_{\kappa ,\beta }$. Let *A* be a neighborhood of $\gamma ^{p}(0)$ in $\Sigma$ such that all unit-speed geodesics starting in *A* orthogonally to $\Sigma$ also exist until at least *T*. We may choose *A* to be compact with $\mu _{\Sigma }(\partial A)=0$ (e.g. as the pre-image of a small, closed ball in $\mathbb {R}^{n-1}$ under a chart of $\Sigma$).

We now show that there exists a neighborhood *U* of *p* such that for any $q\in \tilde{U}:=U\setminus \mathrm {Cut}^{+}(\Sigma )$ we have $b_{\kappa ,\beta }<\tau _{\Sigma }(q)<T$ (which follows immediately from continuity of $\tau _{\Sigma }$) and that the *unique* unit-speed geodesic $\gamma ^{q}$ from $\gamma ^{q}(0)\in \Sigma$ to *q* with $L\left( \gamma ^{q}\right) =\tau _{\Sigma }(q)$ satisfies $\gamma ^{q}(0)\in A$. This is done via contradiction in a similar way to case (1) in the proof of Lemma 3.31: Let $p^{+}\in I^{+}(p)$, then there exists a small neighborhood *U* of *p* such that $\gamma ^{q}(0)\in J^{-}(p^{+})\cap \Sigma$ for all $q\in \tilde{U}$. Assume there exist $p_{j}\in \tilde{U}$ with $p_{j}\rightarrow p$ but $\gamma ^{p_{j}}(0)\notin A$. Then $\gamma ^{p_{j}}\subset J^{-}(p^{+})\cap J^{+}(J^{-}(p^{+})\cap \Sigma )$ and since this set is compact by Remark 3.5 our limit curve Lemma 3.6 shows that there exists $\tilde{\gamma }$ with $p=\tilde{\gamma }(1)$ and $\tilde{\gamma }(0)\ne \gamma ^{p}(0)$ and

$$\begin{aligned} \tau (\tilde{\gamma }(0),p)=L(\tilde{\gamma })\ge \limsup _{j\rightarrow \infty }L(\gamma ^{p_{j}})=\limsup _{j\rightarrow \infty }\tau _{\Sigma }(p_{j})=\tau _{\Sigma }(p), \end{aligned}$$

by continuity of $\tau _{\Sigma }$ (see Lemma 3.7). So $\tilde{\gamma }$ is also maximizing the distance between *p* and $\Sigma$, but this a contradiction since $\tilde{\gamma }\ne \gamma ^{p}$ and $\gamma ^{p}$ was unique since $p\notin \mathrm {Cut}^{+}(\Sigma )$.

We now apply Theorem 1.1 to obtain that

$$\begin{aligned} t\mapsto \frac{\mathrm {vol}\, B_{A}^{+}(t)}{\mathrm {vol}_{\kappa ,\beta }B_{B}^{+}(t)} \end{aligned}$$

is nonincreasing on (0, *T*]. Now the set $\tilde{U}$ from above satisfies $\mu (\tilde{U})\ne 0$ and $\tilde{U}\subset B_{A}^{+}(T)$ but $\tilde{U}\cap B_{A}^{+}(b_{\kappa ,\beta })=\emptyset$, hence $\mathrm {vol}\, B_{A}^{+}(T)>\mathrm {vol}\, B_{A}^{+}(b_{\kappa ,\beta })$. On the other hand, $\mathrm {vol}_{\kappa ,\beta }B_{B}^{+}(t)$ remains constant in *t* for $t\ge b_{\kappa ,\beta }$ by construction of the comparison spaces. But then

$$\begin{aligned} \frac{\mathrm {vol}\, B_{A}^{+}(T)}{\mathrm {vol}_{\kappa ,\beta }B_{B}^{+}(T)}>\frac{\mathrm {vol}\, B_{A}^{+}(b_{\kappa ,\beta })}{\mathrm {vol}_{\kappa ,\beta }B_{B}^{+}(b_{\kappa ,\beta })} \end{aligned}$$

which is a contradiction to $t\mapsto \frac{\mathrm {vol}\, B_{A}^{+}(t)}{\mathrm {vol}_{\kappa ,\beta }B_{B}^{+}(t)}$ being nonincreasing on (0, *T*].

#### *Remark 4.3*

If $\mathbf {Ric}\ge \kappa (n-1)g$ with $\kappa >0$ the mean curvature of $\Sigma$ is irrelevant, hence any globally hyperbolic spacetime satisfying such a curvature bound is necessarily geodesically incomplete: By [21, Thm. 4.5] there exists a smooth metric $g'\succ g$ such that $\left( M,g'\right)$ is globally hyperbolic as well and by [4, Thm. 1.1] there exists a smooth, spacelike Cauchy hypersurface $\Sigma$ for $g'$. This $\Sigma$ is then necessarily acausal ([20, Lemma 14.29 and 14.42]) and FCC (see [25, Rem. 1]) and thus also a smooth, spacelike, acausal, FCC Cauchy hypersurface $\Sigma$ for *g* (by arguments similar to the ones in Lemma 3.12) and $\tau _{\Sigma }\le \frac{\pi }{\sqrt{\kappa }}$: On every compact subset $A\subset \Sigma$ the mean curvature is bounded from above by some $\beta \in \mathbb {R}$ (and this is all that is actually needed to show Theorem 1.1 for this fixed *A*) and since $b_{\kappa ,\beta }\nearrow \frac{\pi }{\sqrt{\kappa }}$ for $\beta \rightarrow \infty$ one arrives at a contradiction by the same construction as in Theorem 4.2. This even shows that $L(\gamma )\le 2\frac{\pi }{\sqrt{\kappa }}$ for any timelike curve $\gamma$ since any inextendible timelike curve must meet $\Sigma$. Of course, the smooth version of this result is well-known and can be proven without this detour [3, Thm. 11.9].

If $\left( M,g\right)$ is not globally hyperbolic, we cannot apply Theorem 1.1 directly, but if $\left( M,g,\Sigma \right)$ satisfies $CCC(\kappa ,\beta )$ with $\kappa ,\beta$ as in Theorem 4.2 and $\Sigma$ is additionally compact we can still use it to prove compactness of the Cauchy development $D^{+}(\Sigma )$.

#### **Lemma 4.4**

Let $\left( M,g,\Sigma \right)$ with $g\in \mathcal {C}^{1,1}$ satisfy $CCC(\kappa ,\beta )$ with $\kappa ,\beta$ as in Theorem 4.2 and $\Sigma$ compact. If $\left( M,g\right)$ is future geodesically complete then $D^{+}(\Sigma )$ is relatively compact.

#### *Proof*

By [14, Thm. A.22 and Prop. A.23] $D(\Sigma )=D(\Sigma )^{\circ }$ is globally hyperbolic, so we may apply Theorem 4.2 to $(D(\Sigma ),g,\Sigma )$, to obtain $\tau _{\Sigma }(p)\le b_{\kappa ,\beta }$ for all $p\in D^{+}(\Sigma )$ and thus $D^{+}(\Sigma )\subset \exp ^{N}([0,b_{\kappa ,\beta }]\cdot \Sigma )$, which is compact. $\square$

The case $\kappa =0$, $\beta <0$ of the previous lemma provides an alternative proof of Hawking’s singularity theorem for $\mathcal {C}^{1,1}$-metrics: Already in the smooth case the proof of Hawking’s singularity theorem splits into two distinct parts, namely an analytic bit, which shows relative compactness of $D^{+}(\Sigma )$, and a part using causality theory. This second part proceeds in the same way whether one deals with smooth or merely $\mathcal {C}^{1,1}$ metrics, so we will not repeat it here (see e.g. [20, Thm. 14.55Aand14.55B] for the smooth case or [14, Thm. 1.1] for the $\mathcal {C}^{1,1}$ proof^1^). Thus we obtain:

#### **Theorem 4.5**

[14, Thm. 1.1] Let $\left( M,g,\Sigma \right)$ with $g\in \mathcal {C}^{1,1}$ satisfy $CCC(\kappa ,\beta )$ with $\kappa ,\beta$ as in Theorem 4.2 and $\Sigma$ compact. Then $\left( M,g\right)$ is future geodesically incomplete.

There seem to be several advantages of this new approach. First, it illustrates the interdependence of the two curvature bounds $\kappa$ and $\beta$ very nicely (see conditions (1) to (3) in Theorem 4.2): The parameter $\beta$ describes the initial focusing ($\beta <0$) or defocusing ($\beta >0$) of geodesics emanating orthogonally to $\Sigma$ (looking at the comparison manifolds in Table 1 we see that $\left| f_{\kappa ,\beta }\right|$ is initially decreasing if $\beta <0$ and increasing if $\beta >0$ and by the formula for the areas in the proof of (16) the same remains true for $\mathrm {area}_{\kappa ,\beta }S_{A}^{+}(t)$), while $\kappa$ describes a global focusing ($\kappa >0$) or defocusing ($\kappa <0$) effect for timelike geodesics. Depending on their relative strength there exists a time $t=b_{\kappa ,\beta }$ where $f_{\kappa ,\beta }$ becomes zero (and the comparison manifold becomes singular) or not. By the volume comparison Theorem 1.1 (and its application in Theorem 4.5) this time gives a universal bound on the maximal time of existence of geodesics starting orthogonally to $\Sigma$ in globally hyperbolic manifolds satisfying the respective curvature bounds. While of course this behavior is also present in the Rauchaudhuri argument used in [14] (and for the smooth case in e.g. [22]) and an analogous argument would also suffice to show cases (1) and (3) from Theorem 4.2, it seems that it is somewhat more explicit in the comparison treatment given here.

Second, while the proof of Theorem 1.1 again relies on approximation arguments, the volume comparison result itself now provides a tool which works directly in $\mathcal {C}^{1,1}$ and allows us to prove other important results (e.g., Theorem 4.2 and Theorem 4.5) without returning to the smooth case.

And, perhaps most importantly, the volume comparison Theorem 1.1 itself is of considerable interest: As already pointed out by the authors of [25], their results are remarkably close to the corresponding Riemannian ones and thus might lend themselves to generalizations of curvature bounds to even lower regularity, a hope that may be strengthened by the $\mathcal {C}^{1,1}$ version of their volume comparison result [25, Thm. 9] proven here.

## Some results from measure theory

To show the measurability of the cut function in Lemma 3.20 we need some tools from measure theory, the main one being the measurable projection theorem (see [5, Thm. III.23]:

### **Theorem A.1**

(Measurable projection)Let $\left( \Omega ,\mathcal {A}\right)$ be a measurable space and *S* a Suslin space. If *G* is measurable in the product $\sigma$-algebra $\mathcal {A}\otimes \mathcal {B}(S)$ (where $\mathcal {B}(S)$ denotes the Borel-$\sigma$-algebra of *S*), then its projection $\mathrm {pr}_{\Omega }(G)\subset \Omega$ is universally measurable.

This statement uses the following definitions (see [5, Def. III.17 and III.21]):

### **Definition A.2**

(*Suslin and polish spaces*) A Suslin space is a Hausdorff topological space that is the continuous image of a Polish space. A Polish space is a separable completely metrizable topological space.

### *Example A.3*

Clearly, $\mathbb {R}$ is Polish, hence also Suslin.

### **Definition A.4**

(*Universal* $\sigma$ *-algebra*) Let $\left( \Omega ,\mathcal {A}\right)$ be a measurable space. Given any finite measure $\mu$ we denote the completion of $\mathcal {A}$ with respect to $\mu$ by $\mathcal {A}_{\mu }$. Then the universal $\sigma$-algebra $\hat{\mathcal {A}}$ is defined as

$$\begin{aligned} \hat{\mathcal {A}}:=\bigcap _{\mu \,\mathrm {finite}}\mathcal {A}_{\mu }. \end{aligned}$$

### *Remark A.5*

If $\mu$ is a $\sigma$-finite measure on $\left( \Omega ,\mathcal {A}\right)$ then there exists an equivalent (i.e., having the same zero-measure sets) measure $\tilde{\mu }$ that is finite. So one has

$$\begin{aligned} \hat{\mathcal {A}}=\bigcap _{\mu \,\sigma -\mathrm {finite}}\mathcal {A}_{\mu }. \end{aligned}$$

This shows that any universally measurable set is measurable with respect to every complete $\sigma$-finite measure $\mu$ on $\left( \Omega ,\mathcal {A}\right)$.

This allows us to show the following:

### **Proposition A.6**

Given a measurable space $\left( \Omega ,\mathcal {A}\right)$, a Suslin space *S*, a measurable function $f:\Omega \times S\rightarrow \mathbb {R}$ (w.r.t. to the product $\sigma$-algebra $\mathcal {A}\otimes \mathcal {B}(S)$ on $\Omega \times S$ and the Borel-$\sigma$-algebra on $\mathbb {R}$) and a set-valued map $F:\Omega \rightarrow \mathcal {P}(S)$ with $\mathrm {graph}(F):=\left\{ \left( \omega ,s\right) \in \Omega \times S:\, s\in F(\omega )\right\} \in \mathcal {A}\otimes \mathcal {B}(S)$, one has that the map $f^{*}:\Omega \rightarrow \bar{\mathbb {R}}$ defined by

$$\begin{aligned} f^{*}(\omega ):=\sup \left\{ f(\omega ,s):\, s\in F(\omega )\right\} \end{aligned}$$

is measurable with respect to the universal $\sigma$-algebra $\hat{\mathcal {A}}$ (and the Borel-$\sigma$-algebra on $\bar{\mathbb {R}}$). $\square$

### *Proof*

It clearly suffices to show that $\left\{ \omega :\, f^{*}(\omega )>r\right\} \in \hat{\mathcal {A}}$ for all $r\in \mathbb {R}$. This follows immediately from

$$\begin{aligned} \left\{ \omega :\, f^{*}(\omega )>r\right\} =\mathrm {pr}_{\Omega }\left( \mathrm {graph}(F)\cap \left\{ (\omega ,s):\, f(\omega ,s)>r\right\} \right) , \end{aligned}$$

measurability of *f*, $\mathrm {graph}(F)\in \mathcal {A}\otimes \mathcal {B}(S)$ and the measurable projection theorem A.1. $\square$

Another statement we need concerns itself with the measurability of the graph of a measurable function (and can, e.g., be found in [23, Prop. 3.1.21])

### **Proposition A.7**

Let $\left( \Omega ,\mathcal {A}\right)$ be a measurable space and *X* a second countable topological space satisfying the $T_{1}$ separation axiom (for every pair of distinct points there exists a neighborhood for each that does not contain the other) with Borel-$\sigma$-algebra $\mathcal {B}(X)$. If $f:\Omega \rightarrow X$ is measurable, then $\mathrm {graph}(f)\in \mathcal {A}\otimes \mathcal {B}(X)$.

### *Proof*

Let $U_{n}$ be a countable basis of open sets, then

$$\begin{aligned} y\ne f(x)\Longleftrightarrow \exists n:\, f(x)\in U_{n}\mathrm {\, and\,}y\notin U_{n}. \end{aligned}$$

So we have

$$\begin{aligned} \mathrm {graph}(f)=\left[ \bigcup _{n}f^{-1}(U_{n})\times U_{n}^{c}\right] ^{c}, \end{aligned}$$

hence it is measurable. $\square$

Furthermore, if the graph of a function between two $\sigma$-finite measure spaces is measurable (and points have measure zero), it has measure zero:

### **Proposition A.8**

Let $\left( \Omega _{1},\mathcal {A}_{1},\mu _{1}\right)$ and $\left( \Omega _{2},\mathcal {A}_{2},\mu _{2}\right)$ be two $\sigma$-finite measure spaces such that $\mu _{2}(\left\{ x\right\} )=0$ for all $\left\{ x\right\} \in \mathcal {A}_{2}$. Assume $f:\Omega _{1}\rightarrow \Omega _{2}$ has measurable graph, i.e. $\mathrm {graph}(f)\in \mathcal {A}_{1}\otimes \mathcal {A}_{2}$, then $\mu _{1}\otimes \mu _{2}(\mathrm {graph}(f))=0$.

### *Proof*

We apply Fubini’s theorem to the characteristic function $\chi _{\mathrm {graph}(f)}$. This gives that for any $x\in \Omega _{1}$ the functions

$$\begin{aligned} \chi _{\mathrm {graph}(f)}(x,.)=\chi _{\{f(x)\}} \end{aligned}$$

from $\Omega _{2}\rightarrow \mathbb {R}$ are measurable, hence $\left\{ f(x)\right\} \in \mathcal {A}_{2}$ for all *x* and so by assumption on $\mu _{2}$ we have $\mu _{2}\left( \left\{ f(x)\right\} \right) =0$. But then Fubini gives

$$\begin{aligned} \mu _{1}\otimes \mu _{2}(\mathrm {graph}(f))=\int _{\Omega _{1}}\int _{\Omega _{2}}\chi _{\{f(x)\}}(y)d\mu _{2}(y)d\mu _{1}(x)=0, \end{aligned}$$

proving the claim. $\square$

Finally, we will state a result concerning images of sets of measure zero under Lipschitz continuous functions on $\mathbb {R}^{n}$ (which can be found, e.g., in [11, Prop. 3.2] for differentiable maps, but the proof only uses the Lipschitz property) that is needed in various proofs of this work.

### **Proposition A.9**

Let $f:\mathbb {R}^{n}\rightarrow \mathbb {R}^{n}$ be Lipschitz continuous. If $A\subset \mathbb {R}^{n}$ has (Lebesgue-)measure zero, then $f(A)\subset \mathbb {R}^{n}$ has (Lebesgue-)measure zero as well.

## Footnotes

1 Note that the future convergence in [14] is the negative of the mean curvature and hence it is bounded from below in the assumptions of [14], Thm. 1.1].
